# Digital finance and corporate breakthrough innovation: Evidence from China

**DOI:** 10.1371/journal.pone.0307737

**Published:** 2024-07-29

**Authors:** Yanmin Shi

**Affiliations:** School of Accounting, Shandong University of Finance and Economics, Jinan, China; American University of the Middle East, KUWAIT

## Abstract

This paper empirically investigates the impact of digital finance on the breakthrough innovation of enterprises with a sample of A-share listed companies in Shanghai and Shenzhen from 2011 to 2022. It is found that digital finance can promote corporate breakthrough innovation, and presents certain structural heterogeneity characteristics. The mechanism test shows that digital finance has the dual attributes of a financing platform and a social platform, which can promote breakthrough innovation by alleviating corporate financing constraints and expanding corporate social networks. Heterogeneity analysis reveals that the role of digital finance in promoting breakthrough innovation is characterized by regional heterogeneity, with digital finance playing a greater role in promoting breakthrough innovation in provinces with a low level of development of the banking sector, provinces with a high level of development of the capital market sector, and the central region. In addition, the degree of firms’ external financing dependence and the degree of product market competition can strengthen the positive effect of digital finance on firms’ breakthrough innovation. This paper enriches the related research on the impact of digital finance on enterprise innovation, and provides theoretical basis and policy insights on how digital finance can better assist the innovation-driven development strategy.

## 1. Introduction

Enterprise innovation is of significant in promoting China’s industrial restructuring and economic transformation and is the core driving force in leading high-quality economic development. The Outline of the Fourteenth Five-Year Plan for the National Economic and Social Development of the People’s Republic of China and the Vision 2035 states that it is necessary to "strengthen the status of enterprises as the main body of innovation, and promote the concentration of all kinds of innovation factors in enterprises." Under the internal and external constraints of increasing uncertainty in the world economic situation and the domestic economy being in a critical period of transforming the growth momentum, the key to solving the problem of the "necklace" of critical technologies and the successful implementation of the innovation-driven development strategy lies in the ability to improve the quality of innovation. Although the quantity of innovation in Chinese enterprises has increased significantly, the quality of innovation still needs to improve. According to the dual innovation theory, enterprise innovation can be categorized into progressive and breakthrough innovation according to the nature of change and the degree of innovation [1]. Progressive innovation refers to minor improvements to existing products along the current path based on existing knowledge. In contrast, breakthrough innovation refers to breaking the boundaries of existing knowledge and utilizing brand-new technologies to create new products. Progressive innovation mainly reflects the quantity of innovation [1]. In contrast, breakthrough innovation can change the existing technological paradigm and expand the original technological boundaries, which is more groundbreaking and disruptive [2], and helps enterprises eliminate the "necklace" dilemma, which is an essential reflection of the quality of innovation [3]. Therefore, in the context of China’s efforts to enhance its core competitiveness and build world-class enterprises, it is of great practical significance to explore how to enhance breakthrough innovation for Chinese enterprises to improve the quality of innovation and gain international competitive advantages.

Breakthrough innovation is a destructive innovation that requires firms to invest more resources and take more significant risks. The innovation outcome is also more uncertain, leading to breakthrough innovation facing more serious financing constraints. Moreover, breakthrough innovation is based on brand-new knowledge, which requires enterprises to conduct extensive knowledge search and exploration [4]. Financial institutions can provide a source of funds for enterprise innovation activities. However, China’s traditional finance has revealed some structural mismatches in supporting enterprise innovation activities, which has led to an apparent “liquidity stratification” phenomenon of financial resources and constrained the potential driving force of financial institutions in the development of innovation, especially breakthrough innovation. In order to make finance better serve the real economy and improve the quality of enterprise innovation, the 20th Party Congress emphasizes “deepening the reform of the financial system.” The digital economy has become the most dynamic, innovative and widely radiating economic form. Traditional finance has taken the opportunity of the booming development of the digital economy to organically integrate with emerging digital technologies such as big data, cloud computing and information technology to form a new financial service model, and digital finance has emerged. Unlike traditional finance, digital finance empowers or upgrades financial products and business processes with the help of technology, characterized by low cost, low threshold and convenient sharing [5]. Among them, big data processing technology can alleviate the problem of information asymmetry in the financial market [6], cloud computing technology can promote the connection between financial subjects and reduces transaction costs to expand and strengthen the communication and exchange between the subjects involved in the financial market; information technology can help to broaden the content and boundaries of financial services and optimizes the allocation of resources. Therefore, digital finance may lay the foundation for breakthrough innovation of enterprises by alleviating financing constraints and enhancing networks relationships.

Studies have shown that digital finance can significantly contribute to corporate innovation [5–8]. For example, Zhao et al. argue that digital finance not only helps firms to increase R&D investment but also can positively affect the quantity and quality of innovations [7]. Zhang et al. demonstrate the positive effect of digital finance on innovations regarding the number of patents filed and granted [8]. Xie and Wu conducted a study using panel data from Chinese provinces and found that digital finance can boost regional R&D conversion rates [5]. Specifically, Yao and Yang conducted a study using a sample of Chinese GEM-listed companies and found that digital finance can stimulate innovation vitality and improve the innovation level of enterprises [6]. Scholars generally agree that digital finance can positively impact enterprise innovation. However, scholars have not yet reached a unanimous conclusion on the incentive mechanism therein, with most scholars agreeing with the mechanism of financing constraint alleviation in digital finance [6–8], and some scholars proposing a mechanism of bank competition improvement [8], and business environment improvement mechanism [7]. The above results provide strong theoretical support and methodological inspiration for this paper to explore how digital finance affects enterprise breakthrough innovation. However, breakthrough innovation belongs to a higher degree of innovation mode, which has similarities and differences with innovation, especially breakthrough innovation, which needs to be based on new knowledge, which determines that digital finance may have heterogeneous effects on enterprise breakthrough innovation through other mechanisms. Unfortunately, there is a lack of a complete analytical framework and empirical findings on the digital finance drive in corporate breakthrough innovation, and only Zhao et al. briefly discusses the differences in the degree of incentives of digital finance on incremental and breakthrough innovations in his study of digital finance to promote the innovation output of firms [7].

Digital finance, as a new financial service model resulting from the fusion of traditional finance and emerging digital technologies, is bound to have the financing attributes of finance and higher efficiency of its financing services on the one hand [9,10], and on the other hand, it also has the attributes of social networking based on emerging digital technologies [11]. Theoretically, financing services can provide the necessary capital for breakthrough innovation, while social networks can provide access to the knowledge needed. Given this, this paper takes A-share listed companies in Shanghai and Shenzhen from 2011 to 2022 as the research sample, empirically analyzes how digital finance affects corporate breakthrough innovation, and further examines its intrinsic mechanism. It has been found that digital finance can promote corporate breakthrough innovation. The mechanism analysis finds that the attributes of the financing platform and social networking platform of digital finance can alleviate the financing constraints and expand the social network of enterprises, positively promoting breakthrough innovation.

Compared with the existing literature, the research contribution of this paper is mainly reflected in the following three aspects: first, empirical analysis of the effect and path of digital finance on corporate breakthrough innovation, which not only expands the research on the impact of digital finance on the behavior of microenterprises, but also enriches the results of the literature on corporate breakthrough innovation; second, the study empirically examines the path of digital finance affecting corporate breakthrough innovation, and found that digital finance can promote corporate breakthrough innovation by alleviating the financing constraints and expanding social networks, which is specific guidance for the further development direction of China’s financial institutions; third, the study finds that in promoting corporate breakthrough innovation, digital finance has a deficiency-complementing effect of delivering charcoal in the snow and an advantageous gain of icing on the cake in the capital market, which provides direct empirical evidence supporting the government’s positive significance of the comprehensive development of the financial market.

## 2. Theoretical and hypotheses

According to the dual innovation theory, incremental innovation is to improve the original product based on existing knowledge [12], while breakthrough innovation requires companies to break the boundaries of established knowledge and make disruptive changes to existing products or services based on exploring new knowledge in order to enter into a completely new technological field [12–14]. It can be seen that breakthrough innovation emphasizes the exploration of new knowledge compared to incremental innovation’s use of existing knowledge, which requires firms to pay higher costs and take more significant risks, as well as more resource support [12]. Therefore, abundant financial support and heterogeneous knowledge allow firms to engage in breakthrough innovation [15]. However, Chinese firms generally need more conditions to carry out breakthrough innovation at present, and it has become an essential way for Chinese firms to enhance their innovation capability by obtaining financial support and heterogeneous knowledge to support their breakthrough innovation behavior [16,17].

Relying on information technology such as big data, cloud computing and blockchain, digital finance has the dual attributes of a financing platform and a social platform. By alleviating financing constraints and expanding social networks, digital finance can lay the foundation of capital and knowledge for corporate breakthrough innovations.

### 2.1 Financing platform attribute of digital finance and corporate breakthrough innovation

From the perspective of alleviating financing constraints, finance is a core component of the enterprise innovation environment, and the adequate supply of finance will inevitably also affect the development of corporate breakthrough innovation activities [18]. However, traditional finance has problems such as insufficient supply and uneven distribution in serving the real economy, which makes enterprises face more serious financing constraints and restricts the development of breakthrough innovation activities. According to the theory of financing constraints, information asymmetry, and transaction costs are the main reasons for financing constraints. At the same time, digital finance can alleviate the financing constraints of enterprises by increasing the quantity of financial resources supply, improving the efficiency of financial resources allocation, and reducing the information asymmetry between enterprises and financial institutions, thus promoting enterprises to carry out breakthrough innovation.

First, digital finance increases the amount of financial resources supplied. Under the current financial system, China’s financial resources rely mainly on the banking sector to provide them. However, because the knowledge assets accumulated by breakthrough innovations are usually intangible [19], the limited collateralized value of intangible assets restricts the use of debt [20]. According to the theory of financing constraints, in the case of the low collateralized value of corporate assets, the amount of financial resources available to the enterprise is limited, and the cost increases, resulting in the breakthrough innovation of the enterprise facing a severe problem of financing constraints. digital finance based on fintech can revitalize existing financial resources outside the formal financial system and bring into the market financial businesses that used to be fragmented, niche and unattended. Moreover, digital finance has a low-cost advantage, and developing a series of financial resources often accompanies its emergence. These are conducive to expanding the financial resource system and increasing the supply of financial resources, which enables enterprises to obtain more financial support at a lower cost and helps them to carry out breakthrough innovation.

Second, digital finance improves the efficiency of financial resource allocation. There are many decentralized small-scale investors in the financial market, and these investors are the long-tail group in the financial market. Traditional finance is confined to technology, cost, etc., and cannot efficiently absorb the long-tail group, resulting in the inefficiency of traditional financial services. Moreover, digital finance, supported by information technology, can process massive data based on low cost and low risk, broaden the boundary of financial services, reach a broader range of tail groups, reduce the threshold and cost of financial services, form a more efficient model of financial service, and reduce the cost of financing for breakthrough innovation of enterprises. In addition, digital finance, as a kind of financial spillover, subverts the traditional credit pricing model through the transparency and informatization of credit, which can force financial institutions to transform and upgrade to a certain extent [15,21] making financial services more compatible with the needs of enterprises, improve the efficiency of financial resource allocation, and provide financial support for the breakthrough innovation of enterprises.

Third, digital finance reduces the information asymmetry between firms and financial institutions. One of the essential functions of financial markets is to assume the role of information matcher between borrowers and lenders, and the key to lending and borrowing lies in the control of risk and credit. According to the information asymmetry theory, compared with the enterprises themselves, the financial sector needs a higher degree of understanding of the credit and risk information of the enterprises. The breakthrough innovation activities are characterized by significant investment, long cycles, and high uncertainty of the results, leading to enterprises facing more significant risks. When the traditional financial sector predicts that enterprises face more significant uncertainty risks, it is often reluctant to conduct a detailed assessment of the capital return on the output of breakthrough innovations, leading to a reduction in the willingness of financial institutions to lend to enterprises and the number of loans. Moreover, digital finance, relying on information technology and big data technology, can dynamically monitor enterprise behavior, capture enterprise behavior data promptly and effectively integrate it, quickly match information between different subjects, and deeply understand and master enterprise risk information [22]. On this basis, digital finance establishes a third-party credit system, conducts a more accurate credit evaluation of enterprises [23], reduces information asymmetry, enhances the willingness of financial institutions to lend money and the amount of money, and lays the financial foundation for corporate breakthrough innovation.

It can be seen that digital finance can play the role of a financing platform by increasing the supply of financial resources, improving the efficiency of financial resource allocation and reducing the information asymmetry between enterprises and financial institutions, thus promoting corporate breakthrough innovations. Therefore, this paper proposes the hypothesis1 as follows:

**H1:** Digital finance can promote corporate breakthrough innovation by acting as a financing platform.

### 2.2 Social networking platform attribute of digital finance and corporate breakthrough innovation

From the perspective of expanding the social networks, which is the sum of a series of strong and weak social relationships owned by the top management of an organization [24], according to the theory of “embeddedness”, all the innovative activities of an enterprise are embedded in the social networks in which it is located. Since breakthrough innovation needs to be based on new heterogeneous knowledge [12,13], according to social networks theory, social networks can provide channels and paths for knowledge exploration. Moreover, digital finance not only has the attribute of a financing platform but also has the attribute of a social platform [11], which can broaden the social networks of enterprises, strengthen the strong social relations of the enterprise and develop the weak social relations of the enterprise, so that the enterprise can more conveniently carry out the exploration and acquisition of knowledge, and then promote the enterprise to carry out breakthrough innovation.

First, strong social relations refer are those multiple social relations based on trust and emotion, where transferring information and resources between networks subjects is more efficient [25]. Using digital payments and digital finance requires enterprises to transact financial assets that require a high level of security through the Internet, which increases trust in society and strengthens solid social relations [26]. Strong social relationships can increase the level of knowledge exchange and sharing among networks subjects, which helps enterprises obtain critical information about technological changes in the industry and market demand, thus giving rise to new ideas and technologies and laying the knowledge base for breakthrough innovation [27]. In addition, solid social relations can also shorten the path of innovation resource transfer so that enterprises with solid social relations can obtain relevant innovation knowledge promptly and gain a first-mover advantage in information, which helps to enhance the willingness and motivation of breakthrough innovation.

Second, a weak social relationship refers to a single relationship based on business, a non-redundant social relationship in which there is a high degree of heterogeneity in knowledge and information among networks subjects [17]. Digital finance is not only a financing platform that provides convenience for participants in financial activities but also a social platform that promotes collaboration between enterprises and other social subjects. Decentralized models such as the “fan economy” can help enterprises continuously expand their social networks, which contributes to weak social relationships [17]. Weak social relations can provide a bridge for network subjects with different knowledge, experience and cultural backgrounds to share and communicate [17], which facilitating knowledge transfer and technology exchange between enterprises and other enterprises, acquires heterogeneous information and knowledge different from the existing knowledge base, and promotes the generation of new knowledge and technology, thus facilitating corporate breakthrough innovation. In addition, weak social relationships have greater openness, more opportunities for cross-border exchanges, and lower knowledge acquisition costs, which can encourage enterprises to search for more partners and acquire more heterogeneous knowledge, thus helping to generate new ideas and promote breakthrough innovation.

It can be seen that digital finance can play the role of a social network platform by strengthening the solid social relations of enterprises and expanding the weak social relations of enterprises, thus promoting enterprises to carry out breakthrough innovation. Therefore, this paper proposes hypothesis H2 as follows:

**H2:** Digital finance can promote corporate breakthrough innovation by acting as a social networking platform.

## 3. Methodology

### 3.1 Sample selection and data sources

This paper selects A-share listed companies in Shanghai and Shenzhen from 2011 to 2022 as samples to study the impact of digital finance on the breakthrough innovation of enterprises, and screens the data in accordance with the following criteria: (1) compared with other industries, the financial industry is relatively special in its business activities, and the preparation of financial statements is quite different from that of enterprises in other industries, therefore, the samples of listed companies in the financial industry are excluded; (2) To avoid the influence of abnormal data of ST and PT companies, the samples of ST, *ST, and PT companies are excluded; (3) Excluding the samples with missing values for key variables. A total of 18179 observations are obtained after screening. Meanwhile, in order to avoid the influence of sample outliers, continuous variables are subjected to Winsorize shrinkage at 1% and 99% quantile.

The patent classification data used in this paper are from the China Economic and Financial Database (CCER), the data on the number of patent applications are from the China Research Data Service Platform database (CNRDS), the data on digital finance are from the China Digital Inclusive Finance Index (provincial level) published by the China Center for Digital Finance Research of Peking University, and the data on company financials, corporate governance, and provinces are from the database of the CSMAR.

### 3.2 Variables

#### Dependent variable

The dependent variable is Enterprise breakthrough innovation. This paper uses the degree of patent dispersion (Break) as a proxy variable for firms’ breakthrough innovation [28]. China’s patent classification adopts the IPC classification of the International Patent Classification List (IPC), which is set according to the technical subject matter, and the form of the IPC classification number is “Part-Major Class-Minor Class-Main Group-Subgroup” with five levels of classification, among which there are eight parts, namely, Part A, B, C, D, E, F, G or H. Each part has one letter, major class has two numbers, minor class has one letter, main group has one letter, and each part has one letter. 1 letter, 2 numbers for major class, 1 letter for minor class, 1–3 numbers for main group, 2–4 numbers for subgroup, the second level of classification is a mandatory supplemental classification based on the patent itself has been mandatorily categorized on the other hand of the subject matter of other classified positions, a total of 131 second level of classification of patents. The degree of patent dispersion is based on the patent classification number of the listed company to calculate the degree of dispersion of the enterprise’s patents under the secondary classification. The specific calculation method refers to Formula (1):

$$Break=1-\sum_{k}^{n}S_{ik}^{2}$$ (1)

where *S*_*ik*_ denotes the proportion of patents of enterprise *i* under the secondary classification *k*. A larger value of Break indicates that the enterprise’s patents are distributed in more decentralized fields, revealing that the enterprise has explored more technological fields, and therefore, a higher degree of breakthrough innovation. Wen et al. [29] and Li and Li [30] used this approach to measure corporate breakthrough innovation. In addition, since breakthrough innovation not only represents that an enterprise has explored in more technological fields, but also represents a higher quality of innovation, this paper also adopts the number of invention patent applications (Patent) as a proxy variable for an enterprise’s breakthrough innovation, which is calculated as the natural logarithm of the number of enterprise’s invention patent applications in the current year +1.

#### Independent variable

The independent variable is Digital finance (Index), Drawing on the studies of Xue and Zha [31] and Wang and Liu [32], this paper takes the Digital Inclusive Finance Index of Chinese Provinces published by the China Digital Finance Research Center of Peking University (from now on referred to as the Peking University Digital Finance Index) as a proxy variable for digital finance, and logarithmizes the index.

Theoretically, digital finance is a multidimensional concept involving multiple indicators of different dimensions. The Peking University Digital Finance Index not only takes into account the population and geography covered by digital finance but also the depth of its use while also taking into account horizontal comparability at the regional level and vertical comparability in the time dimension from a dynamic perspective, so that the Peking University Digital Finance Index is better able to measure the degree of development of digital finance. Peking University Digital Finance Index covers 33 specific indicators in three dimensions: coverage, depth, and digitization. Among them, the coverage reflects the extent to which digital finance ensures that users get corresponding financial services, including account coverage rate, payment, and money fund; the depth reflects the actual use of digital financial services by users, including credit, insurance, investment, and credit; and the digitization reflects the advantages of low-cost and low-threshold advantage of digital financial services, including mobility, affordability, credibility, and Facilitation [33]. The details are shown in Table 1.

**Table 1:** The Peking University Digital Finance indicator system.

Level 1 dimension	Level 2 dimension		Specific indicators
**Coverage**	Account coverage rate		Number of Alipay accounts per 10,000 people
			Proportion of Alipay card-bound users
			Average number of bank cards tied to each Alipay account
	Payment		Number of payments per person
			Payment per person
			High-frequency (fifty or more times active per year) number of active users / one or more times active per year
	Money fund		Number of Yu Ebao per person
			Amount of Yu Ebao per person
			Number of Yu Ebao per 10,000 Alipay users
**Depth**	Credit business	Personal	Internet consumer loans per 10,000 adult Alipay users
			Number of loans per person
			Amount of loans per person
		Small business	Internet small business loans per 10,000 adult Alipay users
			Average number of loans per small business
			Average amount of loans per small business
	Insurance		Number of insured users per 10,000 Alipay users
			Number of insurance per person
			Amount of insurance per person
	Investment		Number of people participating in Internet investment and finance management per 10,000 Alipay users
			Number of investments per person
			Amount of investment per person
	Credit		Number of times per person that credit for natural persons is called up
			Number of users using credit-based services per 10,000 Alipay users (including finance, accommodation, travel, social, etc.)
**Digitization**	Mobility		Percentage of mobile payment transactions
			Percentage of mobile payment amount
	Affordability		Average lending rate for micro and small operators
			Average personal lending rate
	Credibility		Percentage of Chanting Payment Transactions
			Percentage of Chanting Payment Amount
			Sesame Credit Pledge-Free Transactions as a Percentage (compared to all cases requiring a deposit)
			Sesame Credit Pledge-Free Amounts as a Percentage (compared to all cases where a deposit is required)
	Facilitation		Percentage of payments made by users using two-dimensional codes
			Percentage of amount paid by users on two-dimensional codes

#### Control variables

This paper controls for a number of variables that may affect firms’ breakthrough innovations, Firm size (Size): measured using the natural logarithm of firms’ total assets; Gearing (Lev): measured using the ratio of total liabilities to total assets; Return on net assets (Roe): measured using the ratio of net income to the average balance of equity interest; Growth (Grow): measured using operating income growth rate; Current ratio (Flow): measured by the ratio of current assets to current liabilities; Asset liquidity (Liqui): measured by the ratio of current assets to total assets; Equity concentration (Top1): measured by the proportion of shares held by proxy shareholders; Two positions in one (Dual): if the chairman of the board of directors and the general manager are held by the same person, it takes the value of 1, otherwise, it takes the value of 0; Region level of economic development (Deve): measured using the natural logarithm of per capita GDP by province, and controlling for year (Year) and industry (Industry) fixed effects. Specific variable definitions are detailed in Table 2.

**Table 2:** Definition of variables.

Variables	Description of variables
Break	Calculated from Eq (1)
Patent	Natural logarithm of the number of patent applications for inventions + 1
Index	Natural logarithm of the Digital Inclusive Financial Development Index (provincial level)
Size	Natural logarithm of total assets
Lev	Total liabilities/total assets
Roe	Net profit/average balance of shareholders’ equity
Grow	Revenue growth rate
Flow	Current assets/current liabilities
Liqui	Current assets/total assets
Top1	Shareholding ratio of the largest shareholder
Dual	Dummy variable. Takes the value of 1 if the chairman and general manager are the same person, 0 otherwise
Deve	Natural logarithm of GDP per capita by province

### 3.3 Model definition

Referring to Li et al. [34] and Li and Zhou [35], this paper uses the least squares method (OLS) to test model (2) in order to explore the relationship between digital finance and corporate breakthrough innovation:

$$Y_{i,t}=\alpha_{0}+\alpha_{1}{Index}_{p,t}+\alpha_{2}{Controls}_{p,i,t}+\sum Year+\sum Industry+\varepsilon_{i,t}$$ (2)

Where, *p* represents province, *i* represents enterprise, and *t* represents year, *Y*_*i*,*t*_ represents corporate breakthrough innovation, including *Break*_*i*,*t*_ and *Patent*_*i*,*t*_. *Break*_*i*,*t*_ denotes the degree of patent dispersion of firm *i* in year *t*, and *Patent*_*i*,*t*_ denotes the number of invention patents applied by enterprise *i* in year *t*. *Index*_*p*,*t*_ denotes the digital finance index of province *p* in year *t*; *Controls*_*p*,*i*,*t*_ denotes firm-and-province-level control variables. Since variables that do not vary with time and industry also impact the regression results, to control for the impact of these omitted factors, this paper controls for the time dummy variable *Year* and the industry dummy by variable *Indusry*. *ε*_*i*,*t*_ denotes the random error term. If digital finance can promote the corporate breakthrough innovation, it is expected that the regression coefficient *α*_1_ of *Index*_*p*,*t*_ in model (2) will be significantly positive.3.4 Descriptive statistics.

Table 3 reports the descriptive statistics of the variables. The results show that the minimum value of the degree of dispersion of patent distribution of enterprises is 0.000, the maximum value is 1.000, and the standard deviation is 0.340, indicating that there are large differences in the degree of dispersion of patent distribution of the sample enterprises, with some enterprises exploring in more technological fields, with patents involved in more dispersed fields, and some enterprises exploring in only one or a few technological fields. The mean value of the number of enterprise invention patent applications is 2.220, the median is 2.197, the minimum value is 0.000, the maximum value is 6.236, and the standard deviation is 1.420, which indicates that at least half of the enterprises in the sample did not reach the mean value of the number of invention patent applications of the sample enterprises, and there is also a big difference in the number of invention patent applications of sample enterprises, which indicates that the breakthrough of Chinese enterprises innovation level is low, and the quality of enterprise innovation needs to be improved. The minimum value of digital finance is 3.426, the maximum value is 6.071, and the standard deviation is 0.550. The digital finance index of the province with the highest level of digital finance development is about 11 times higher than that of the province with the lowest level of digital finance development, which indicates that there are large differences in the level of digital finance development in different provinces.

**Table 3:** Descriptive statistics of variables.

Variables	Obs	Mean	Std	Min	Median	Max
Break	15,565	0.690	0.340	0.000	0.833	1.000
Patent	15,565	2.220	1.420	0.000	2.197	6.236
Index	15,565	5.421	0.550	3.426	5.595	6.071
Size	15,565	22.092	1.239	20.069	21.885	26.179
Lev	15,565	0.399	0.196	0.050	0.389	0.869
Roe	15,565	0.072	0.111	-0.489	0.074	0.354
Grow	15,565	0.161	0.323	-0.445	0.112	1.830
Flow	15,565	2.639	2.635	0.379	1.770	17.141
Liqu	15,565	0.585	0.177	0.136	0.596	0.925
Top1	15,565	34.040	14.273	8.482	32.139	73.331
Dual	15,565	0.305	0.461	0.000	0.000	1.000
Deve	15,565	10.986	0.463	10.040	10.942	12.013

### 3.4 Correlation coefficients

Table 4 reports the correlation coefficients between the variables. The results show that the correlation coefficients between digital finance and the degree of dispersion of enterprise patent distribution and the number of enterprise invention patent applications are both significantly positive, indicating that digital finance can promote enterprises to explore in more technological fields and the quality of the patents they apply for is higher, which preliminarily proves the research hypotheses of this paper, H1 and hypothesis H2.The correlation coefficients between the control variables are all less than 0.7, which indicates that there is no serious multicollinearity.

**Table 4:** Pearson correlation.

	Break	Patent	Index	Size	Lev	Roe	Grow	Flow	Liqu	Top1	Dual	Deve
Break	1.0000											
Patent	0.379***	1.0000										
Index	0.096***	0.136***	1.0000									
Size	0.154***	0.431***	0.125***	1.0000								
Lev	0.096***	0.207***	0.0050	0.533***	1.0000							
Roe	0.032***	0.096***	-0.016**	0.096***	-0.178***	1.0000						
Grow	-0.0090	0.063***	-0.035***	0.021**	0.014*	0.311***	1.0000					
Flow	-0.079***	-0.149***	-0.087***	-0.366***	-0.674***	0.090***	-0.045***	1.0000				
Liqu	0.063***	0.029***	-0.040***	-0.266***	-0.190***	0.152***	0.033***	0.371***	1.0000			
Top1	0.021***	0.0060	-0.082***	0.170***	0.041***	0.118***	-0.0070	-0.021***	-0.0100	1.0000		
Dual	-0.0100	-0.050***	0.055***	-0.180***	-0.141***	0.020**	0.039***	0.110***	0.078***	-0.022***	1.0000	
Deve	0.049***	0.103***	0.587***	0.136***	0.0090	0.0040	-0.0040	-0.0040	0.0110	-0.035***	0.037***	1.0000

Note

* P<0.1

** P<0.05

*** P<0.01.

## 4. Empirical analysis

### 4.1 Baseline regression analysis

Table 5 reports the regression results of the model (2). The first two columns present the regression results of digital finance on the degree of decentralization of firms’ patent distribution. Column (1) is the regression result without controlling the variables that can affect both digital finance and the degree of patent distribution dispersion, while column (2) is the regression result with the addition of control variables, and the regression coefficients of digital finance on the degree of patent distribution dispersion of firms are all positive and significant at the 1% confidence level. The last two columns are the regression results of digital finance on the number of enterprise invention patent applications. Column (3) is the regression result without controlling variables that can affect digital finance and the number of invention patent applications at the same time, and the regression coefficients of digital finance on the number of enterprise invention patent applications are positive and significant at the 1% confidence level. Column (4) is the regression result of adding control variables, the regression coefficient of digital finance on the number of enterprise invention patent applications is positive and significant at 5% confidence level. The regression results in Table 4 show that digital finance can not only encourage enterprises to explore in more technological fields, but also the quality of patent applications is higher, digital finance can promote enterprises to carry out breakthrough innovation, and the research hypothesis H1 and hypothesis H2 is proved.

**Table 5:** The regression results of digital finance and corporate breakthrough innovation.

	Corporate breakthrough innovation			
	Break		Patent	
	(1)	(2)	(3)	(4)
Index	0.0553*** (3.036)	0.0909*** (3.040)	0.4225*** (5.806)	0.2324** (2.258)
Size		0.0521*** (19.045)		0.6277*** (57.077)
Lev		-0.0279 (-1.261)		-0.3812*** (-4.878)
Roe		0.0962*** (3.595)		0.5440*** (5.633)
Grow		-0.0266*** (-2.909)		0.0766** (2.358)
Flow		-0.0075*** (-4.811)		-0.0276*** (-5.591)
Liqu		0.1591*** (8.592)		0.8657*** (12.407)
Top1		0.0001 (0.428)		-0.0045*** (-6.440)
Dual		0.0035 (0.612)		0.0286 (1.382)
Deve		-0.0237** (-2.252)		-0.0169 (-0.451)
Cons	0.1397* (1.799)	-0.9442*** (-9.940)	-0.8039*** (-2.716)	-13.7483*** (-39.457)
Year	Yes	Yes	Yes	Yes
Industry	Yes	Yes	Yes	Yes
Obs	15565	15565	15565	15565
Adj_R^2^	0.0928	0.1281	0.1001	0.3355

Note

***, **, and * denote, in turn, significance at the 1%, 5%, and 10% confidence levels; numbers in parentheses are t-values, and regression results are based on robust standard errors.

Among the control variables, the regression coefficients of enterprise size on the degree of patent distribution dispersion and the number of invention patent applications are all significantly positively correlated at the 1% confidence level, indicating that the larger the enterprise, the higher the level of risk-taking, the easier it is to explore in a variety of technological fields, the greater the number of invention patents, and the higher the level of breakthrough innovation. The regression coefficient of gearing ratio on the number of invention patent applications of enterprises is significantly negative at 1% confidence level, indicating that the higher the gearing ratio is, the higher the long-term debt repayment pressure of enterprises, and the enterprises will reduce the breakthrough innovation behaviors in order to avoid risks. The regression coefficients of return on net assets on the degree of patent distribution dispersion and the number of invention patent applications are significantly positive at 1% confidence level, indicating that the higher the efficiency of the enterprise in utilizing its own capital, the easier it is to carry out breakthrough innovation. The regression coefficients of enterprise growth on the degree of patent distribution dispersion are significantly negative at 1% confidence level, and the regression coefficients of the number of invention patent applications are significantly positive at 5% confidence level, indicating that the higher the growth of the enterprise, the less likely to expand the field of technology, and the more likely to deepen the existing technology and improve the quality of innovation. The regression coefficients of asset liquidity on the degree of patent distribution dispersion and the number of invention patent applications are both significantly positive at the 1% confidence level, indicating that the more liquid assets, the more likely that enterprises will carry out breakthrough innovation. The regression coefficients of the shareholding ratio of the first major shareholder on the degree of patent distribution dispersion are positive but not significant, and the regression coefficients of the number of invention patent applications are significantly negative at 1% confidence level, which indicates that the higher the shareholding ratio of the first major shareholder is, the stronger the motivation of the major shareholder to infringe on the interests of the minority shareholders, and the more unfavorable it is for the enterprise to improve the quality of innovation.

### 4.2 Endogeneity test

#### 4.2.1 Instrumental variables approach

Since breakthrough innovation is a firm-level behavior and digital finance is a provincial-level variable, it is less likely that firms’ breakthrough innovation will have a reverse causal effect on digital finance, but there may still be a problem of correlation between the main explanatory variables and the disturbance term. This paper addresses this endogeneity issue with the help of instrumental variables approach.

Referring to Jiang et al. [36], Wang and Guo [37], and Qu and Zhu [38], this paper uses Internet penetration (Internet) in each province as an instrumental variable for digital finance [39]. The selection of instrumental variables must satisfy the two conditions of relevance and exogeneity. Specifically, in this paper, the instrumental variables should be related to digital finance without direct correlation with the breakthrough innovation of enterprises. On the one hand, the development of digital finance cannot be separated from the popularization and application of the Internet. The Internet provides conditions for financial institutions to apply digital technologies such as big data and cloud computing, which can effectively promote the development of digital finance, so the Internet penetration rate as an instrumental variable of digital finance satisfies the relevance condition [36,37]; on the other hand, Internet penetration influences the behavior of enterprises in a longer path, and thus Internet penetration is unlikely to directly affect the corporate breakthrough innovation. Therefore, Internet penetration as an instrumental variable of digital finance satisfies the condition of exogeneity [38]. The P-value of Hausman test for the exogeneity test is 0.0210, which rejects the original hypothesis that all the explanatory variables are exogenous, suggesting that the model is indeed endogenous and suitable for the endogeneity test using the instrumental variable method. The Kleibergen-Paap rk LM F-value of the unidentifiable test is 1625.839, which is greater than the critical value of 10, rejecting the original hypothesis of unidentifiable. The Cragg-Donald Wald F-value of the weak instrumental variable test is 2184.423, which is greater than the critical value of 10, rejecting the original hypothesis that the instrumental variables are weakly instrumental variable. Thus, it proves that the Internet penetration as a digital instrumental variable of finance is valid.

The test results of the two-stage least squares (2SLS) regression with Internet penetration as the instrumental variable are shown in Table 6. Column (1) shows that the regression coefficient of Internet penetration on digital finance is significantly positive, which meets the correlation requirement of instrumental variables; column (2) and column (3) show that the regression coefficients of digital finance on the degree of dispersion of enterprise patent distribution and the number of invention patent applications are both significantly positive at the confidence level of 1%, which indicates that after the endogeneity test of the instrumental variable with Internet penetration, the positive effect of digital finance on enterprises’ breakthrough innovation is still significant. After the endogeneity test of the variables, the positive effect of digital finance on promoting breakthrough innovation by enterprises is still significant, indicating that the previous benchmark regression results have good robustness.

**Table 6:** The regression results of instrumental variables approach.

	Phase I	Phase II	
	Index	Break	Patent
	(1)	(2)	(3)
Internet	0.4839*** (46.2990)		
Index		0.2631*** (3.3649)	1.9591*** (6.5965)
Size	0.0010 (1.3540)	0.0521*** (19.0513)	0.6278*** (56.8771)
Lev	-0.0048 (-0.7994)	-0.0262 (-1.1808)	-0.3637*** (-4.6092)
Roe	-0.0005 (-0.0691)	0.0955*** (3.5694)	0.5373*** (5.5115)
Grow	-0.0012 (-0.5669)	-0.0268*** (-2.9397)	0.0742** (2.2646)
Flow	0.0009* (1.8109)	-0.0077*** (-4.9027)	-0.0292*** (-5.8030)
Liqu	0.0054 (1.0638)	0.1577*** (8.5212)	0.8516*** (12.1232)
Top1	0.0000 (0.4315)	0.0001 (0.4039)	-0.0045*** (-6.4590)
Dual	0.0051*** (3.5106)	0.0018 (0.3058)	0.0113 (0.5382)
Deve	0.1632*** (59.7712)	-0.0734*** (-3.1431)	-0.5151*** (-5.8070)
Cons	1.8804*** (61.8088)	-1.0790*** (-9.8258)	-15.0999*** (-36.6445)
Year	Yes	Yes	Yes
Industry	Yes	Yes	Yes
Obs	15565	15565	15565
Hausman	P = 0.0210		
Kleibergen-Paap rk LM	F = 1625.839		
Cragg-Donald Wald	F = 2184.423		
Adj_R^2^	0.9760	0.1260	0.3233

Note

***, **, and * denote, in turn, significance at the 1%, 5%, and 10% confidence levels; numbers in parentheses are t-values, and regression results are based on robust standard errors.

#### 4.2.2 Control other possible missing variables

First, financial development can reduce monitoring costs, weaken principal-agent problems, improve the availability of external financing, and have an impact on firms’ technological innovation. Therefore, the impact of digital finance on firms’ breakthrough innovation may be due to differences in regional financial development levels. In order to alleviate the endogeneity problem caused by possible omitted variables, this paper controls for the level of financial development in each province to test the previous benchmark regression again. In this paper, the level of bank development in each province is used as a proxy variable for the level of financial development in each province and is included in the control variables of the benchmark regression model (2). Among them, the level of bank development in each province is measured by the financial related ratio (Bank) and financial depth (Loan), with the financial related ratio being the ratio of the sum of deposit and loan balances of financial institutions to GDP, and the financial depth being the ratio of loan balances of financial institutions to GDP.

Second, while controlling for industry-and-year-fixed effects in the previous section, some characteristics of a firm’s province may also impact corporate breakthrough innovation. Although we control for province-level variables (Deve, Bank, Loan), there may still be some unobservable variables that can affect corporate breakthrough innovation. Therefore, this paper further controls for province-fixed effects and their joint fixed effects with industry. In addition, to control for the effects of unobservables that do not vary with industry and time, this paper also controls for industry and year joint fixed effects.

The regression results after controlling for other possible omitted variables are shown in Table 7. The first two columns report regression results controlling for the level of financial development in each province, and the regression coefficients of digital finance on corporate breakthrough innovation are both significantly positive at the 1% confidence level. The last two columns report regression results controlling for province-fixed effects and their joint fixed effects with industry and joint fixed effects with industry and year, and the regression coefficients of digital finance on corporate breakthrough innovation are positive. The regression results controlling for other possible omitted variables are generally consistent with the previous benchmark regression results, indicating that digital finance can still play a positive role in corporate breakthrough innovations after controlling for factors at the province level that may affect corporate breakthrough innovation. It proves the robustness of the findings of the benchmark study in this paper.

**Table 7:** The regression results of controlling of possible omitted variables in the province.

	Control for province’s financial development		Fixed (joint fixed) effects	
	Break	Patent	Break	Patent
	(1)	(2)	(3)	(4)
Index	0.1073*** (3.4381)	0.3144*** (2.9241)	0.0543* (1.749)	0.4189 (0.146)
Size	0.0546*** (18.0401)	0.6231*** (51.5263)	0.0400*** (16.545)	2.0946*** (9.373)
Lev	-0.0239 (-1.0020)	-0.3516*** (-4.2043)	0.0054 (0.287)	0.8076 (0.463)
Roe	0.0937*** (3.2388)	0.6627*** (6.2169)	0.0834*** (3.702)	-0.7528 (-0.361)
Grow	-0.0367*** (-3.6257)	0.0737** (2.0985)	-0.0164** (-2.224)	1.8821*** (2.754)
Flow	-0.0072*** (-4.4111)	-0.0259*** (-5.1516)	-0.0037*** (-2.853)	1.0030*** (8.361)
Liqu	0.1598*** (7.8656)	0.8391*** (11.1640)	0.1235*** (7.693)	-1.2227 (-0.824)
Top1	0.0001 (0.3696)	-0.0051*** (-6.7066)	0.0004** (2.332)	-0.0741*** (-4.934)
Dual	0.0043 (0.6857)	0.0350 (1.5459)	-0.0014 (-0.300)	0.1284 (0.287)
Deve	0.0076 (0.6045)	-0.1705*** (-3.7145)	0.0441 (1.344)	-4.8782 (-1.607)
Bank	-0.0215*** (-3.6394)	0.1476*** (6.6329)		
Loan	0.0173 (1.0325)	-0.3897*** (-6.2085)		
Cons	-1.3667*** (-11.4840)	-12.2648*** (-27.4886)	-0.9986** (-2.554)	47.6903 (1.319)
Industry	Yes	Yes	Yes	Yes
Year	Yes	Yes	Yes	Yes
Province	No	No	Yes	Yes
Province-Industry	No	No	Yes	Yes
Industry-Year	No	No	Yes	Yes
Obs	13324	13324	17488	17488
Adj_R^2^	0.1263	0.3354	0.2557	0.1956

Note

***, **, and * denote, in turn, significance at the 1%, 5%, and 10% confidence levels; numbers in parentheses are t-values, and regression results are based on robust standard errors.

#### 4.2.3 Control characteristics of the technical field

Byun et al. argued that the number and concentration of a firm’s technological fields may impact its technological spillovers, which may affect its breakthrough innovations [12]. For this reason, referring to Byun et al. [12], this paper includes the number of fields covered by patents filed by firms (Techn) and their concentration (HHI) as control variables in the model (2).

Table 8 shows the regression results controlling for the characteristics of technological fields. The first two columns are regression results controlling for the number of fields covered by the patents applied by enterprises. The last two columns are regression results controlling for the concentration of technological fields covered by the patents applied by enterprises. The coefficients of digital finance on corporate breakthrough innovations are significantly positive, which is in line with the baseline regression results, indicating that the conclusions of this paper’s research are robust.

**Table 8:** The regression results of controlling characteristics of the technical field.

	Break	Patent	Break	Patent
	(1)	(2)	(3)	(4)
Index	0.1072*** (17.314)	0.1702*** (8.987)	0.0879*** (14.327)	0.0861*** (4.223)
Size	0.0139*** (5.010)	0.2903*** (26.701)	0.0266*** (11.311)	0.4962*** (45.941)
Lev	0.0513** (2.563)	0.0387 (0.576)	0.0333* (1.702)	-0.2220*** (-3.051)
Roe	0.0078 (0.321)	0.0298 (0.352)	-0.0066 (-0.278)	0.0233 (0.254)
Grow	0.0278*** (3.430)	0.1518*** (5.271)	0.0211*** (2.620)	0.0661** (2.179)
Flow	-0.0042*** (-2.656)	-0.0211*** (-4.916)	-0.0044*** (-2.857)	-0.0279*** (-6.266)
Liqu	0.1552*** (9.848)	0.8529*** (15.103)	0.1736*** (11.487)	1.2324*** (20.585)
Top1	0.0001 (0.510)	-0.0059*** (-9.753)	0.0001 (0.492)	-0.0064*** (-9.895)
Dual	0.0057 (1.132)	0.0184 (1.051)	0.0064 (1.286)	0.0533*** (2.866)
Deve	-0.0032 (-0.483)	-0.0179 (-0.802)	0.0037 (0.576)	0.0198 (0.836)
Techfiled	0.0009*** (26.865)	0.0095*** (53.335)		
HHItf			-0.3741*** (-25.117)	-2.0703*** (-43.133)
Cons	-0.2223*** (-2.885)	-5.2439*** (-17.500)	-0.3899*** (-5.589)	-9.2026*** (-31.356)
Industry	Yes	Yes	Yes	Yes
Year	Yes	Yes	Yes	Yes
Obs	15574	15574	15574	15574
Adj_R^2^	0.1086	0.4397	0.1433	0.3651

Note

***, **, and * denote, in turn, significance at the 1%, 5%, and 10% confidence levels; numbers in parentheses are t-values, and regression results are based on robust standard errors.

### 4.3 Robustness tests

#### 4.3.1 Tobit regression

From the previous descriptive statistical analysis, it can be seen that the degree of patent distribution dispersion and the number of invention patent applications are all greater than 0, and there are data that are zero. The breakthrough innovation indicators show the phenomenon of zero-value accumulation, and the regression with OLS model may have some bias. Therefore, this paper re-regresses the benchmark model (2) with the Tobit model.

The regression results using the Tobit model are shown in the first two columns of Table 9, the regression coefficients of digital finance on the degree of patent distribution dispersion and the number of invention patent applications are significantly positive at least at the 5% confidence level, which is consistent with the previous benchmark regression results, indicating that there is a positive effect of digital finance on the breakthrough innovations of enterprises regardless of whether the OLS model or the Tobit model is used. The main conclusions of this paper are not affected by the choice of regression model, and the regression results are relatively robust.

**Table 9:** The regression results of Tobit model and independent variable lagged one period.

	Tobit		Independent variable lagged one period	
	Break	Patent	Break	Patent
	(1)	(2)	(3)	(4)
Index	0.1049*** (3.2500)	0.2863** (2.4851)		
L.Index			0.0823*** (13.449)	0.1686*** (7.419)
Size	0.0573*** (18.0556)	0.6671*** (58.8529)	0.0482*** (18.933)	0.6652*** (58.115)
Lev	-0.0423* (-1.7382)	-0.4409*** (-5.0790)	-0.0156 (-0.701)	-0.3724*** (-4.397)
Roe	0.1102*** (3.6506)	0.5994*** (5.5535)	0.0710*** (2.804)	0.5296*** (5.404)
Grow	-0.0296*** (-3.0260)	0.0832** (2.3892)	0.0014 (0.162)	-0.0045 (-0.131)
Flow	-0.0094*** (-5.7054)	-0.0331*** (-5.6116)	-0.0065*** (-3.539)	-0.0340*** (-5.525)
Liqu	0.1818*** (8.6726)	0.9395*** (12.5881)	0.1577*** (8.574)	0.9027*** (11.842)
Top1	0.0001 (0.2345)	-0.0053*** (-6.9953)	-0.0000 (-0.071)	-0.0050*** (-6.638)
Dual	0.0041 (0.6339)	0.0230 (0.9906)	0.0036 (0.659)	0.0193 (0.879)
Deve	-0.0276** (-2.3425)	-0.0421 (-1.0035)	0.0149** (2.112)	0.0130 (0.473)
Cons	-1.1481*** (-10.8602)	-14.7760*** (-39.2550)	-1.2021*** (-13.633)	-14.3992*** (-41.000)
Industry	Yes	Yes	Yes	Yes
Year	Yes	Yes	Yes	Yes
Obs	15565	15565	13731	13731
Pseudo R^2^	0.1152	0.1067		
Adj_R^2^			0.1222	0.3504

Note

***, **, and * denote, in turn, significance at the 1%, 5%, and 10% confidence levels; numbers in parentheses are t-values, and regression results are based on robust standard errors.

#### 4.3.2 Independent variable lagged one period

Although there is unlikely a reverse causality endogeneity problem of digital finance on corporate breakthrough innovations, there may be a certain lag in the promotion of digital finance on corporate breakthrough innovations due to the long breakthrough innovation cycle. Drawing on Xie and Wu [5] and Xue et al. [31], this paper regresses digital finance one period lagged.

The regression results of the independent variable lagged one period are shown in the last two columns of Table 9. The regression coefficient of digital finance on corporate breakthrough innovation is significantly positive at the 1% level, which is consistent with the benchmark regression results, indicating that this paper’s research conclusions have good robustness.

#### 4.3.3 Substitution of variable measures

This paper further employs the method of replacing the measurement of variables for robustness testing. First, the measure of digital finance is changed by normalizing the digital finance index (Index_Normal) and regressing the benchmark model (2).

The regression results of replacing the measure of digital finance are shown in columns (1) and (2) of Table 10, where the regression coefficients of digital finance on the degree of patent distribution dispersion and the number of invention patent applications are significantly positive at least at the 10% confidence level, which is consistent with the previous benchmark regression results, suggesting that replacing the measurement of the digital finance variable does not change the results of the baseline regression and that the conclusion that digital finance drives firms to breakthrough innovation is robust.

**Table 10:** The regression results of replacing the measurement of variables.

	Digital financial		corporate breakthrough innovation			
	Break	Patent	Citation	Kind	Techp	Cita90
	(1)	(2)	(3)	(4)	(5)	(6)
Index_Normal	0.0459* (1.9081)	0.3296*** (3.7104)				
Index			0.4875*** (4.5039)	0.1732** (2.1881)	-0.0295** (-2.032)	1250.9035* (1.916)
Size	0.0522*** (19.0679)	0.6283*** (57.1813)	0.6091*** (56.2793)	0.3112*** (35.9085)	-0.0101*** (-9.186)	1845.3190*** (17.525)
Lev	-0.0284 (-1.2813)	-0.3803*** (-4.8643)	-0.7704*** (-9.9931)	-0.1044* (-1.7471)	-0.0021 (-0.230)	-1726.2517*** (-5.200)
Roe	0.0956*** (3.5738)	0.5383*** (5.5732)	0.1273 (1.5085)	0.3945*** (5.3459)	0.0038 (0.347)	1103.4306** (2.271)
Grow	-0.0263*** (-2.8753)	0.0783** (2.4071)	-0.0248 (-0.8733)	-0.1501*** (-6.2926)	0.0094*** (2.641)	-679.6134*** (-5.249)
Flow	-0.0074*** (-4.7534)	-0.0273*** (-5.5286)	-0.0422*** (-7.4523)	-0.0261*** (-7.1067)	0.0017*** (2.951)	-20.9519 (-1.409)
Liqu	0.1585*** (8.5505)	0.8583*** (12.3129)	0.9736*** (14.2304)	0.6667*** (12.9943)	0.0129* (1.694)	1795.5612*** (4.682)
Top1	0.0001 (0.4094)	-0.0045*** (-6.4909)	-0.0048*** (-6.7477)	0.0013** (2.4764)	0.0000 (0.290)	-4.5784 (-1.119)
Dual	0.0036 (0.6305)	0.0252 (1.2153)	0.0265 (1.2517)	0.0297* (1.9313)	0.0044* (1.918)	534.0197*** (5.509)
Deve	-0.0252 (-1.5999)	-0.1490*** (-2.5795)	0.1022*** (2.6488)	-0.0614** (-2.1390)	0.0213 (1.343)	1193.2146** (2.039)
Cons	-0.6021*** (-3.5667)	-11.6233*** (-18.5939)	-15.5360*** (-44.4930)	-6.4285*** (-23.7199)	0.9454*** (5.090)	-59255.3304*** (-8.500)
Firm	Yes	Yes	Yes	Yes	Yes	Yes
Year	Yes	Yes	Yes	Yes	Yes	Yes
Obs	15565	15565	17498	15570	16747	13142
Adj_R^2^	0.1277	0.3359	0.3109	0.2521	0.0355	0.1560

Note

***, **, and * denote, in turn, significance at the 1%, 5%, and 10% confidence levels; numbers in parentheses are t-values, and regression results are based on robust standard errors.

Second, this paper also adopts the method of changing the way of measuring the variables of corporate breakthrough innovation for robustness testing. Referring to the common practice in the current literature, this paper adopts the number of citations of the enterprise’s authorized patents (Citation) and the number of the enterprise’s patents entering the second-level classification (Kind) as the proxy variables for the enterprise’s breakthrough innovation [40,41], respectively. Among them, the specific calculation of the number of citations of the enterprise’s authorized patents. The number of patent citations can reflect the technical importance of a firm’s innovations, and patents with high citations usually represent path-breaking critical innovations [42]. The number of enterprise patents entering the second classification level is calculated as the natural logarithm of the number of enterprise patents crossing the second level of IPC classification. The number of patents entering the secondary classification reflects the number of technological fields the firm enters. If a firm enters more technological fields, it indicates that it creates new knowledge with low relevance to the stock of knowledge. Therefore, these two indicators can also better measure corporate breakthrough innovation. In addition, referring to Byun et al. [12], this paper also adopts technology proximity (Techp) and patent citation ranking (Cita90) as proxy variables for breakthrough innovation. Technological proximity is the proximity between the technological field covered by a firm’s new patent application and the technological field covered by existing patents, reflecting the extent of the firm’s deviation from technological research. The larger the value of technological proximity, the smaller the degree of breakthrough innovation of the enterprise. Patent citation ranking refers to the number of patents in the top 10% of citation distribution among all patents applied by an enterprise in that year and the total number of patents applied by the enterprise in that year, reflecting the degree of recognition of patents. The larger the value of patent citation ranking, the higher the degree of breakthrough innovation of the enterprise.

The regression results of replacing the measurement of corporate breakthrough innovation variables are shown in columns (3) and (6) of Table 10. The coefficients of digital finance on the number of citations of enterprises’ licensed patents is significantly positive at the 1% confidence level. The coefficients of the number of enterprises’ patents entering the second level of classification is significantly positive at 5% confidence level, the coefficients of the technology proximity are significantly negative at the 5% level, and the coefficients of the ranking of patent citations are significantly positive at the 10% level, which is consistent with the baseline regression results, indicating that replacing the measurement of breakthrough innovation variables will not change the results of the baseline regression, and the previous findings are robust. In summary, whether replacing the measure of digital finance or replacing the measure of firms’ breakthrough innovation, the regression results are consistent with the benchmark regression results, suggesting that the finding that digital finance drives firms to engage in breakthrough innovation is well robust.

## 5. Mechanism analysis of financing constraints and social networks

As mentioned in the previous theoretical analysis, digital finance can promote the breakthrough innovation of enterprises through the following paths: first, from the perspective of alleviating financing constraints, digital finance can increase the quantity of financial resources supply, improve the efficiency of financial resources allocation and reduce the information asymmetry between banks and enterprises, so as to alleviate the financing constraints faced by enterprises, and then promote the breakthrough innovation of enterprises. Secondly, from the perspective of expanding social networks, digital finance can strengthen the strong social relationship and develop the weak social relationship of enterprises, expand the social networks of enterprises, and then promote the breakthrough innovation of enterprises. This paper establishes model (3) to test whether digital finance promotes corporate breakthrough innovation by alleviating financing constraints and expanding social networks.

$$Y_{i,t}=\gamma_{0}+\gamma_{1}{Index}_{p,t}+\gamma_{2}{{Index}_{p,i,t}*M_{i,t}+\gamma_{3}M_{i,t}+\gamma_{4}Controls}_{p,i,t}+\sum Industry+\sum Year+\varepsilon_{p,i,t}$$ (3)

The dependent variable (*Y*_*i*,*t*_) in model (3) represents the corporate breakthrough innovation (*Break*_*i*,*t*_, *Patent*_*i*,*t*_). *M*_*i*,*t*_ are the mechanism variables, including financing constraints and social networks. Among them, financing constraints are measured by the KZ index [43,44], where a more extensive KZ index means a greater degree of financing constraints faced by the firm, and vice versa, a more minor degree of financing constraints faced by the firm. The social networks is measured by the network formed through the connection of directors [45], and is measured by the degree of centrality (Degree), which is calculated as follows: *Degree*_*i*,*t*_ = ∑_*j*_*X*_*ji*_/(*n*−1), where *i* denotes a specific enterprise participating in the construction of the social networks, *j* denotes the enterprises other than enterprise *i* in the same year, *X*_*ji*_ denotes the connectivity between enterprises, and *n* denotes the total number of enterprises participating in the construction of the social networks in a specific year.

The regression results of the mechanism analysis of the role of digital finance on corporate breakthrough innovation are shown in Table 11. The mechanism variable in columns (1) and (2) is financing constraint, and the regression coefficients of the interaction term between digital finance and financing constraints are significantly negative at the 10% and 1% levels, respectively, which indicate that digital finance can promote enterprises to make breakthrough innovations by alleviating financing constraints. The mechanism variable in columns (3) and (4) is social networks, and the regression coefficients of the interaction term between digital finance and social networks are significantly negative at the 5% and 1% levels, respectively, indicating that digital finance can promote corporate breakthrough innovation through expanding social networks.

**Table 11:** The regression results of mechanisms analysis of financing constraints and social networks.

	Financing constraints		Social networks	
	Break	Patent	Break	Patent
	(1)	(2)	(3)	(4)
Index	0.1026*** (3.247)	0.1957* (1.794)	0.1242*** (3.695)	0.3161*** (4.913)
KZ	0.0156 (1.346)	0.0860** (2.216)		
Index*KZ	-0.0039* (-1.849)	-0.0200*** (-2.826)		
Deg			0.0083** (2.500)	0.0262** (2.005)
Index*Deg			-0.0014** (-2.272)	-0.0043* (-1.791)
Size	0.0481*** (16.769)	0.6276*** (54.085)	0.0508*** (18.117)	0.5537*** (44.607)
Lev	0.0859*** (3.813)	-0.2518*** (-3.019)	-0.0293 (-1.322)	-0.4441*** (-5.414)
Roe	0.0832*** (2.759)	0.7569*** (6.968)	0.0944*** (3.526)	0.1935* (1.882)
Grow	-0.0241** (-2.494)	0.0945*** (2.791)	-0.0260*** (-2.837)	0.1868*** (5.375)
Liqu	0.1146*** (6.239)	0.7683*** (11.016)	0.1581*** (8.535)	1.3715*** (19.702)
Top1	0.0000 (0.174)	-0.0045*** (-6.279)	0.0001 (0.547)	-0.0069*** (-9.114)
Dual	0.0033 (0.561)	0.0252 (1.187)	0.0044 (0.779)	0.0520** (2.350)
Deve	-0.0275** (-2.524)	-0.0198 (-0.511)	-0.0222** (-2.100)	-0.0413 (-1.517)
Flow			-0.0073*** (-4.637)	-0.0373*** (-7.299)
Cons	-0.8968*** (-8.895)	-13.6561*** (-36.938)	-1.1367*** (-8.707)	-11.7093*** (-23.649)
Industry	Yes	Yes	Yes	Yes
Year	Yes	Yes	Yes	Yes
Obs	14847	14847	15565	15565
Adj_R^2^	0.1266	0.3373	0.1287	0.2249

Note: (1)

***, **, and * denote, in turn, significance at the 1%, 5%, and 10% confidence levels; numbers in parentheses are t-values, and regression results are based on robust standard errors. (2) In the mechanisms analysis of financing constraints, Flow was removed due to multicollinearity.

## 6. Further analysis

### 6.1 Different dimensions of digital finance

This paper uses the Digital Inclusive Finance Index of each province released by the Digital Finance Research Center of Peking University as a proxy indicator of digital finance in each province. Based on the data of Ant Gold Service transaction accounts, the Digital Inclusive Finance Index synthesizes the characteristics of traditional financial services and Internet services, and portrays the level of China’s digital finance development in three first-level dimensions: breadth of coverage, depth of use, and degree of digital support services. Among them, the breadth of digital financial coverage mainly examines the coverage of digital finance from three aspects: the number of Alipay accounts, the proportion of Alipay-bound cards, and the number of bank cards bound to each Alipay account; the depth of use of digital finance measures the actual use of digital finance from the aspects of payment business, insurance business, and money fund services; the degree of digital support services examines the degree of digitization of digital finance in terms of mobility, convenience, affordability and creditworthiness, and is a concentration of Internet technology in traditional financial services. The breadth of digital finance coverage is a precondition, the depth of use reflects the actual use of digital finance, and the degree of digital support services can be regarded as a potential condition. In order to investigate whether the three dimensions of breadth of coverage, depth of use and degree of digital support services have differentiated impacts on breakthrough innovations of enterprises, this paper replaces the digital finance indexes in the baseline model (2) with the breadth of coverage index (Cover), the depth of use index (Usage), and the degree of digital support services index (Digit), respectively, to further test the impacts of the breadth of coverage, depth of use and degree of digital support services on breakthrough innovations of enterprises.

The regression results of the breadth of digital financial coverage, the depth of use and the degree of digital support services on enterprise breakthrough innovation are shown in Table 12. The regression coefficients of the breadth of digital financial coverage on the degree of dispersion of enterprise patent distribution and the number of invention patent applications are significantly positive at the confidence level of 10% and 5% respectively, indicating that the breadth of digital financial coverage has a positive effect on the breakthrough innovation of enterprises, and the wider the scope of digital financial coverage, the more conducive to promoting breakthrough innovation of enterprises; the regression coefficient of the depth of use of digital finance on the degree of dispersion of enterprise patent distribution and the number of invention patent applications are both significantly positive at the confidence level of 1%. The regression coefficients of the number of applications are all significantly positive at the 1% confidence level, indicating that the depth of the use of digital finance can significantly promote the breakthrough innovation of enterprises, and the deeper the degree of the use of digital finance by enterprises, the more conducive to their breakthrough innovation; the regression coefficients of the degree of digital payment services on the degree of dispersion of the distribution of patents by enterprises and the number of applications for patents for invention are all negative but not significant, indicating that the degree of digital support services does not have a significant effect on the breakthrough innovation of enterprises. breakthrough innovation of enterprises does not have a significant effect. Further, in terms of the size of regression coefficients, the regression coefficient of the breadth of digital financial coverage on enterprise breakthrough innovation is smaller than the regression coefficient of the depth of digital financial use on enterprise breakthrough innovation. This indicates that the actual use of digital financial services by enterprises has a greater impact on their breakthrough innovation than the coverage of digital finance. This may be because the breadth of digital financial coverage focuses on the quantitative supply of digital financial services, while the depth of digital financial use focuses more on the effective demand for digital financial services. Therefore, although both the supply and the actual demand for digital financial services can significantly affect the breakthrough innovation of enterprises, the actual demand for digital financial services has a greater impact on the breakthrough innovation of enterprises, which suggests that only the actual use of digital financial services by enterprises can improve their innovation capability.

**Table 12:** The regression results of different dimensions of digital finance.

	Break			Patent		
	(1)	(2)	(3)	(4)	(5)	(6)
Cover	0.0303* (1.8184)			0.1830** (2.1119)		
Usage		0.1396*** (5.5811)			0.4351*** (4.8856)	
Digit			-0.0294 (-1.0899)			-0.0125 (-0.1364)
Size	0.0521*** (19.0301)	0.0524*** (19.1486)	0.0521*** (19.0338)	0.6278*** (57.1188)	0.6286*** (57.1598)	0.6277*** (57.0484)
Lev	-0.0286 (-1.2926)	-0.0271 (-1.2230)	-0.0291 (-1.3127)	-0.3825*** (-4.8939)	-0.3781*** (-4.8352)	-0.3836*** (-4.9078)
Roe	0.0966*** (3.6093)	0.0925*** (3.4513)	0.0961*** (3.5884)	0.5433*** (5.6262)	0.5322*** (5.5029)	0.5446*** (5.6397)
Grow	-0.0267*** (-2.9175)	-0.0261*** (-2.8609)	-0.0266*** (-2.9067)	0.0772** (2.3748)	0.0780** (2.3987)	0.0769** (2.3662)
Flow	-0.0075*** (-4.7941)	-0.0074*** (-4.7211)	-0.0074*** (-4.7517)	-0.0274*** (-5.5451)	-0.0272*** (-5.4994)	-0.0274*** (-5.5469)
Liqu	0.1601*** (8.6356)	0.1568*** (8.4793)	0.1604*** (8.6473)	0.8653*** (12.4056)	0.8582*** (12.3151)	0.8679*** (12.4289)
Top1	0.0001 (0.4238)	0.0001 (0.5121)	0.0001 (0.4374)	-0.0045*** (-6.4813)	-0.0045*** (-6.3815)	-0.0045*** (-6.4313)
Dual	0.0041 (0.7222)	0.0024 (0.4152)	0.0046 (0.8078)	0.0286 (1.3803)	0.0246 (1.1886)	0.0310 (1.5007)
Deve	-0.0100 (-1.0868)	-0.0410*** (-4.1018)	0.0025 (0.3991)	-0.0657 (-1.1205)	-0.0855** (-2.3872)	0.0502** (2.1802)
Cons	-0.8485*** (-9.2114)	-0.9884*** (-10.5363)	-0.7602*** (-5.5324)	-12.4154*** (-19.4190)	-13.9259*** (-40.1577)	-13.5185*** (-27.5058)
Firm	Yes	Yes	Yes	Yes	Yes	Yes
Year	Yes	Yes	Yes	Yes	Yes	Yes
Obs	15565	15565	15565	15565	15565	15565
Adj_R^2^	0.1277	0.1294	0.1276	0.3355	0.3363	0.3353

Note

***, **, and * denote, in turn, significance at the 1%, 5%, and 10% confidence levels; numbers in parentheses are t-values, and regression results are based on robust standard errors.

### 6.2 Particular digital finance tools

In order to more accurately portray the impact of digital finance on corporate breakthrough innovation, this paper further deconstructs the digital finance indexes to explore how particular digital financial tools affect corporate breakthrough innovation in six dimensions: Payment, Insurance, Monetary, Investment, Credit, and Investigation, respectively.

The regression results of particular digital financial tools on corporate breakthrough innovation are shown in Table 13. The regression coefficients of Payment, Monetary, and Credit on corporate breakthrough innovation are significantly positive at the 1% level, indicating that Payment, Monetary, and Credit are important tools for digital finance to promote corporate breakthrough innovation. The regression coefficient of Insurance on the degree of patent distribution dispersion of enterprises is significant at 1% level, and the regression coefficient on the number of invention patent applications of enterprises is positive but not significant; the regression coefficient of Investigation on the degree of patent distribution dispersion of enterprises is positive but not significant, and the regression coefficient on the number of invention patent applications of enterprises is positive at 1% level, indicating that digital finance can promote corporate breakthrough innovation to a certain extent through Insurance and Investigation. The regression coefficient of Investment on corporate breakthrough innovation is positive but insignificant, indicating that the Investment of digital finance does not show the promotion effect on corporate breakthrough innovation.

**Table 13:** The regression results of particular digital finance tools.

**Panel A**						
	**Break**	**Patent**	**Break**	**Patent**	**Break**	**Patent**
	(1)	(2)	(3)	(4)	(5)	(6)
Payment	0.2513*** (4.818)	0.8583*** (4.298)				
Insurance			0.1454*** (2.838)	0.1135 (0.594)		
Monetary					0.2438*** (4.761)	1.0079*** (5.373)
Size	0.0543*** (12.354)	0.6623*** (37.646)	0.0539*** (12.284)	0.6609*** (37.518)	0.0538*** (12.265)	0.6607*** (37.583)
Lev	-0.0595 (-1.621)	-0.5226*** (-3.944)	-0.0568 (-1.543)	-0.5240*** (-3.949)	-0.0588 (-1.602)	-0.5185*** (-3.918)
Roe	0.1150*** (2.706)	0.6546*** (4.357)	0.1172*** (2.760)	0.6672*** (4.427)	0.1142*** (2.688)	0.6486*** (4.318)
Grow	-0.0405*** (-2.957)	0.0042 (0.086)	-0.0394*** (-2.871)	0.0075 (0.152)	-0.0409*** (-2.989)	0.0019 (0.038)
Flow	-0.0126*** (-4.086)	-0.0439*** (-4.527)	-0.0123*** (-3.998)	-0.0439*** (-4.544)	-0.0127*** (-4.113)	-0.0442*** (-4.562)
Liqu	0.1923*** (6.371)	1.0366*** (9.180)	0.1941*** (6.410)	1.0546*** (9.304)	0.1920*** (6.365)	1.0304*** (9.142)
Top1	-0.0001 (-0.455)	-0.0061*** (-5.455)	-0.0001 (-0.488)	-0.0060*** (-5.432)	-0.0001 (-0.395)	-0.0060*** (-5.398)
Dual	-0.0106 (-1.160)	0.0104 (0.313)	-0.0090 (-0.983)	0.0193 (0.581)	-0.0098 (-1.079)	0.0114 (0.344)
Deve	-0.0589*** (-3.520)	-0.1966*** (-3.217)	-0.0161 (-1.281)	0.0072 (0.156)	-0.0517*** (-3.312)	-0.2133*** (-3.796)
Cons	-1.4522*** (-7.317)	-15.7955*** (-20.953)	-1.4552*** (-5.380)	-14.1360*** (-14.043)	-1.4615*** (-7.271)	-16.2874*** (-21.894)
Industry	Yes	Yes	Yes	Yes	Yes	Yes
Year	Yes	Yes	Yes	Yes	Yes	Yes
Obs	6167	6167	6167	6167	6167	6167
Adj_R^2^	0.1191	0.3301	0.1168	0.3278	0.1191	0.3312
**Panel B**						
	**Break**	**Patent**	**Break**	**Patent**	**Break**	**Patent**
	(1)	(2)	(3)	(4)	(5)	(6)
Investment	0.0426 (1.089)	0.1850 (1.257)				
Credit			0.2009*** (5.123)	0.9329*** (6.270)		
Investigation					0.0218 (1.216)	0.2001*** (2.989)
Size	0.0536*** (12.201)	0.6600*** (37.454)	0.0544*** (12.386)	0.6634*** (37.734)	0.0538*** (12.253)	0.6610*** (37.536)
Lev	-0.0586 (-1.594)	-0.5174*** (-3.898)	-0.0605* (-1.648)	-0.5254*** (-3.973)	-0.0600 (-1.634)	-0.5192*** (-3.922)
Roe	0.1182*** (2.784)	0.6649*** (4.408)	0.1140*** (2.683)	0.6452*** (4.313)	0.1165*** (2.741)	0.6451*** (4.278)
Grow	-0.0393*** (-2.866)	0.0082 (0.165)	-0.0407*** (-2.979)	0.0019 (0.038)	-0.0397*** (-2.892)	0.0053 (0.108)
Flow	-0.0125*** (-4.071)	-0.0437*** (-4.526)	-0.0127*** (-4.103)	-0.0442*** (-4.543)	-0.0126*** (-4.103)	-0.0441*** (-4.571)
Liqu	0.1967*** (6.488)	1.0497*** (9.258)	0.1970*** (6.531)	1.0504*** (9.325)	0.1982*** (6.556)	1.0541*** (9.317)
Top1	-0.0001 (-0.428)	-0.0060*** (-5.423)	-0.0001 (-0.420)	-0.0060*** (-5.426)	-0.0001 (-0.443)	-0.0061*** (-5.455)
Dual	-0.0078 (-0.857)	0.0197 (0.592)	-0.0108 (-1.187)	0.0056 (0.171)	-0.0082 (-0.897)	0.0155 (0.466)
Deve	-0.0096 (-0.566)	-0.0424 (-0.661)	-0.0514*** (-3.407)	-0.2412*** (-4.423)	-0.0014 (-0.117)	-0.0411 (-0.979)
Cons	-0.8666*** (-5.526)	-13.8368*** (-24.228)	-1.2066*** (-7.223)	-15.4287*** (-24.886)	-0.8270*** (-5.518)	-13.6913*** (-25.036)
Industry	Yes	Yes	Yes	Yes	Yes	Yes
Year	Yes	Yes	Yes	Yes	Yes	Yes
Obs	6167	6167	6167	6167	6167	6167
Adj_R^2^	0.1158	0.3279	0.1194	0.3325	0.1158	0.3288

Note

***, **, and * denote, in turn, significance at the 1%, 5%, and 10% confidence levels; numbers in parentheses are t-values, and regression results are based on robust standard errors.

### 6.3 Moderating effect

#### 6.3.1 Enterprises’ dependence on external financing

Unlike general investment, innovation investment is characterized by significant investment, long lead time, and high uncertainty, leading to a more severe information asymmetry between firms and external investors. Moreover, to avoid imitation by competitors, firms are reluctant to show their innovation plans to outsiders [46], further exacerbating the degree of information asymmetry. Breakthrough innovation is a higher degree of innovation mode. Therefore, the degree of information asymmetry between firms that engage in breakthrough innovation and external investors is higher. According to the theory of financing constraints, the sources of funds for firms’ investments are categorized into endogenous and external financing, and information asymmetry can lead to differences in internal and external financing costs. Guariglia and Liu [47] argued that information asymmetry causes power imbalance in transactions, leading to high external financing costs and subjecting firms to financing constraints, thus restricting the development of breakthrough innovation [48,49]. It can be seen that the process of digital finance influencing corporate breakthrough innovation may be affected by the extent of firms’ reliance on external financing. Rajan & Zingales conducted a systematic study of the relationship between financial development and economic growth using cross-country data and found that industrial sectors that are relatively more dependent on external financing grow faster in countries with well-developed financial markets [50]. As externally finance-dependent firms face more severe financing constraints, with the increase in financial resources and the reduction in information asymmetry brought about by the development of digital finance, there is more room for improvement in breakthrough innovations of firms with a high degree of external finance dependence compared to firms with a low degree of external finance dependence. In order to test how the degree of external financing dependence affects the relationship between digital finance and breakthrough innovation of enterprises, this paper adds the variable of the degree of enterprises’ external financing dependence (Depend) and its cross-multiplier term with digital finance (Index*Depend) in the baseline regression model (2). Meanwhile, in order to avoid the impact of variable covariance on the regression results, this paper centers digital finance and the degree of corporate external financing dependence. Referring to Hsu et al.’s study [51], the median of external financing needs of all enterprises in dichotomous industries in different provinces in different years is used to express the degree of enterprises’ external financing dependence. The specific calculation method is as follows:

$${depend}_{i,j,k,t}=({capi}_{i,j,k,t}-{cash}_{i,j,k,t}+{rd}_{i,j,k,t})/({capi}_{i,j,k,t}+{rd}_{i,j,k,t})$$ (4)

Where *cash*_*i*,*j*,*k*,*t*_ is calculated as follows:

$${cash}_{i,j,k,t}={prof}_{i,j,k,t}+{disc}_{i,j,k,t}-{\Delta inv}_{i,j,k,t}-{rec}_{i,j,k,t}+{pay}_{i,j,k,t}$$ (5)

In Eq (4), *capi*_*i*,*j*,*k*,*t*_ represents the capital investment expenditure of the enterprise, and the specific estimation method is as follows: ${capi}_{i,j,k,t}=the\;current\;year^{\prime }s\;net\;fixed\;asset\;value-(1-depreciation\;rate)*previous\;year^{\prime }s\;net\;fixed\;asset\;value.{rd}_{i,j,k,t}$ denotes the amount of R&D investment of the enterprise, *prof*_*i*,*j*,*k*,*t*_ denotes the net profit of the enterprise, *disc*_*i*,*j*,*k*,*t*_ denotes the amount of depreciation in the current year, Δ*inv*_*i*,*j*,*k*,*t*_ denotes the amount of increase of the enterprise’s inventory, *rec*_*i*,*j*,*k*,*t*_ represents the accounts receivable of the enterprise, and *pay*_*i*,*j*,*k*,*t*_ represents the accounts payable of the enterprise.

The regression results of the effect of the degree of enterprise external financing dependence on the relationship between digital finance and enterprise breakthrough innovation are shown in Table 14. The regression coefficients of the cross-multipliers of the degree of dependence on external financing on the degree of dispersion of patent distribution and the number of invention patent applications of enterprises are both significantly positive at the 1% confidence level, indicating that digital finance has a greater effect on the enhancement of breakthrough innovations of enterprises with a high degree of dependence on external financing.

**Table 14:** The regression results of enterprises’ dependence on external financing.

	Break	Patent
	(1)	(2)
Index	0.0525** (2.0199)	0.2829*** (2.8919)
Index*Depend	0.0002*** (4.4431)	0.0006*** (4.1839)
Depend	0.0000*** (4.5361)	0.0002*** (6.9648)
Size	0.0529*** (19.2201)	0.6331*** (57.7003)
Lev	-0.0264 (-1.1931)	-0.3668*** (-4.7107)
Roe	0.0893*** (3.3310)	0.4988*** (5.1887)
Grow	-0.0271*** (-2.9711)	0.0739** (2.2814)
Flow	-0.0073*** (-4.6542)	-0.0262*** (-5.3293)
Liqu	0.1577*** (8.5231)	0.8544*** (12.2910)
Top1	0.0001 (0.7069)	-0.0043*** (-6.1655)
Dual	-0.0001 (-0.0250)	0.0048 (0.2328)
Deve	-0.0210 (-1.2857)	-0.0971 (-1.6049)
Cons	-0.6322*** (-3.4191)	-12.0569*** (-17.4835)
Industry	Yes	Yes
Year	Yes	Yes
Obs	15565	15565
Adj_R^2^	0.1299	0.3390

Note

***, **, and * denote, in turn, significance at the 1%, 5%, and 10% confidence levels; numbers in parentheses are t-values, and regression results are based on robust standard errors.

#### 6.3.2 Competition in product markets

Currently, several literatures have confirmed the existence of a significant effect of competition on firms’ innovation, for example, Arrow points out that as firms increase their degree of monopoly over the market, firms make profits through monopoly rather than through innovation, which reduces the firms’ incentives to innovate [52]; however, Aghion found that there is an inverted U-shaped relationship between the “flight effect” and the “Schumpeterian effect” of competition on corporate innovation [53].

China’s favorable innovation environment and development opportunities drive the incentive effect of market competition on enterprises’ investment in innovation. As market competition intensifies, enterprises’ financing costs, capital needs and cash flow sensitivity increase, endogenous financing capacity decreases, and reliance on external funding increases. Therefore, as market-based competition intensifies, the positive effect of digital finance on breakthrough corporate innovation will become more prominent. In addition, market competition can improve the quality of corporate information disclosure, which is conducive to the firm’s understanding and access to information about other firms’ relevant technological innovations and learning and borrowing, which will strengthen the positive effect of digital finance on breakthrough innovations of enterprises.

In order to test the impact of market competition on the relationship between digital finance and corporate breakthrough innovation, this paper adds product market competition (Comp, Epcm) and its interaction term with digital finance (Index*Comp, Index*Epcm) to the benchmark regression model (2). Among them, the degree of competition in the product market is measured by two indicators: the degree of competition in the industry (Comp) and enterprise market power (Epcm), the degree of competition in the industry (Comp) is measured by the number of enterprises in the industry, and the enterprise market power (Epcm) is measured by the "enterprise Lerner index—industry Lerner index of the industry to which the enterprise belongs to in the current year", controlling for the differences in the structure of the industry. Comp is measured by the number of firms in the industry, and Epcm is measured by the “firm Lerner index—industry Lerner index for the industry to which the firm belongs to in the current year”, controlling for differences in industry structure. Enterprise market power represents the bargaining power and market position of an enterprise, and the greater the market power of an enterprise, the weaker the degree of market competition it faces. Meanwhile, in order to avoid the influence of variable covariance on the regression results, the degree of competition in the digital finance and product markets is centralized.

The regression results of the influence of product market competition degree on the relationship between digital finance and enterprise breakthrough innovation are shown in Table 15. Columns (1) and (2) show how the degree of industry competition affects the positive relationship between digital finance and enterprise breakthrough innovation. It can be seen that the regression coefficient of the interaction term between digital finance and industry competition on the degree of enterprise patent distribution dispersion is significantly positive at 1% confidence level. The regression coefficient for the number of invention patent applications of enterprises is significantly positive at the confidence level of 5%, indicating that the degree of industry competition can strengthen the positive effect of digital finance on enterprises’ breakthrough innovation. Columns (3) and (4) show how enterprise market power affects the positive relationship between digital finance and enterprise breakthrough innovation. It can be seen that the regression coefficient of the interaction term of digital finance and enterprise market power on the degree of enterprise patent distribution dispersion is significantly negative at the 1% confidence level, and the regression coefficient on the number of enterprise invention patent applications is negative but not significant. It shows that the smaller the market power of enterprises, the stronger the positive impact of digital finance on enterprises’ breakthrough innovation. Based on the regression results in Table 13, the degree of product market competition can generally strengthen the promoting role of digital finance on enterprise breakthrough innovation. The higher the degree of product market competition, the more prominent the role of digital finance on enterprise breakthrough innovation.

**Table 15:** The regression results of competition in the product market.

	Degree of competition in the industry		Corporate market power	
	Bi	Patent	Bi	Patent
	(1)	(2)	(3)	(4)
Index	0.1171*** (3.773)	0.2854*** (2.646)	0.0951*** (3.159)	0.2254** (2.171)
Index*Comp	0.0001*** (2.946)	0.0004** (2.171)		
Index*Epcm			-0.2398*** (-4.564)	-0.0917 (-0.519)
Comp	-0.0003*** (-3.418)	-0.0003 (-0.857)		
Epcm			-0.0789** (-2.325)	0.3023*** (2.593)
Size	0.0522*** (19.069)	0.6286*** (57.214)	0.0516*** (18.588)	0.6279*** (56.181)
Lev	-0.0268 (-1.206)	-0.3898*** (-4.989)	-0.0184 (-0.797)	-0.3622*** (-4.478)
Roe	0.0957*** (3.572)	0.5345*** (5.538)	0.0546* (1.696)	0.7215*** (6.311)
Grow	-0.0265*** (-2.891)	0.0733** (2.255)	-0.0242*** (-2.578)	0.0971*** (2.942)
Flow	-0.0075*** (-4.784)	-0.0280*** (-5.687)	-0.0078*** (-4.881)	-0.0237*** (-4.704)
Liqu	0.1587*** (8.578)	0.8635*** (12.385)	0.1674*** (8.923)	0.8433*** (11.945)
Top1	0.0001 (0.469)	-0.0044*** (-6.292)	0.0001 (0.543)	-0.0046*** (-6.562)
Dual	0.0032 (0.571)	0.0282 (1.366)	0.0030 (0.523)	0.0371* (1.791)
Deve	-0.0285*** (-2.655)	-0.0250 (-0.660)	-0.0240** (-2.268)	-0.0180 (-0.477)
Cons	-0.6157*** (-3.656)	-12.6356*** (-20.815)	-0.4352*** (-2.641)	-12.5192*** (-21.153)
Industry	Yes	Yes	Yes	Yes
Year	Yes	Yes	Yes	Yes
Obs	15565	15565	15393	15393
Adj_R^2^	0.1286	0.3360	0.1294	0.3372

Note

***, **, and * denote, in turn, significance at the 1%, 5%, and 10% confidence levels; numbers in parentheses are t-values, and regression results are based on robust standard errors.

### 6.4 Heterogeneity analysis

#### 6.4.1 The level of financial development

Financial repression in China is at a high level, and the process of improvement is slow, which prevents financial demand from being effectively matched. The mismatch between the quantity and quality of financial services and the effective demand for financial is one of the key reasons why digital finance has been able to develop rapidly in China. Digital finance has upgraded and transformed traditional finance with the help of emerging digital technologies such as big data, cloud computing, and blockchain, and to a certain extent, it has exerted the "inclusive effect" of incremental supplementation [36,54]. Therefore, in regions where traditional finance is relatively weak, digital finance may play a greater role in facilitating breakthrough innovation for enterprises. On the other hand, digital finance is a form of financial services implemented by the traditional financial sector with the help of technology, and technological innovation is a potential driving force for the development of digital finance. However, at the same time, it can also bring financial risks and affect the financial system’s stability [55]. The contagious nature of financial risks may lead to further contagion in the capital market. In contrast, more developed capital markets are more risk-resilient, thus helping to mitigate the adverse effects of digital finance. Therefore, digital finance may play a greater role in facilitating breakthrough innovation for firms in capital markets with higher levels of development.

In order to test whether there is a differential impact of digital finance on breakthrough innovation of enterprises under different financial endowment conditions, this paper examines the level of development of traditional finance from two aspects: the banking sector and the capital market sector. Among them, the development level of the banking sector is measured by the ratio of the total loan size of each province to its GDP size [56]; and the development level of the capital market sector is measured by the ratio of the total market value of stocks outstanding at the end of the year to the total GDP of each province [57]. On this basis, the annual median of the level of development of the banking sector was used as a dividing criterion, and samples above the annual median were categorized into the group with a high level of development of the banking sector (Bank_High), and samples below the annual median were categorized into the group with a low level of development of the banking sector (Bank_Low); similarly, using the annual median of the level of development of the capital market sector as a dividing criterion, the sample above the annual median is classified as the group with a high level of development of the capital market sector (Market_High) and the sample below the annual median is classified as the group with a low level of development of the capital market sector (Market_Low). Subgroup regressions are performed for the baseline model (2).

The results of the grouped regressions of digital finance on firms’ breakthrough innovations under different financial endowments are shown in Table 16. Panel A shows the impact of digital finance on firms’ breakthrough innovations under different levels of development in the banking sector. It can be seen that in the group with low level of development of banking sector, the regression coefficients of digital finance on the degree of dispersion of enterprise patent distribution and the number of invention patent applications are positive and significant at 1% confidence level, in the group with high level of development of banking sector, the regression coefficients of digital finance on the degree of dispersion of enterprise patent distribution and the number of invention patent applications are positive but insignificant, and the test of coefficient of difference between the groups shows that, no matter it’s the degree of dispersion of enterprise patent distribution or the number of invention patent applications, there is a significant difference of regression coefficients of digital finance at 5% confidence level between the two groups of samples with high and low level of development of banking sector. Panel B shows the impact of digital finance on firms’ breakthrough innovations under different levels of capital market sector development. It can be seen that the regression coefficient of digital finance on the degree of dispersion of firms’ patent distribution is significantly positive at 1% confidence level in the group with a high level of development of the capital market sector, and the regression coefficient of digital finance on the degree of dispersion of firms’ patent distribution is positive but not significant in the group with a low level of development of the capital market sector; the regression coefficients of digital finance on the number of invention patent applications filed by firms are significantly positive in both the high and low sample groups for the level of development of the capital market sector, but the test for the difference in the coefficients between the groups reveals that the regression coefficients of digital finance in the two sample groups are significantly different at the 5% confidence level.

**Table 16:** The regression results of the level of financial development heterogeneity.

**Panel A Level of development of the banking sector**				
	**Break**		**Patent**	
	**Bank_Low**	**Bank_High**	**Bank_Low**	**Bank_High**
	(1)	(2)	(3)	(4)
Index	0.1611*** (3.4470)	0.0290 (0.8443)	0.6199*** (3.8962)	0.1134 (0.8750)
Size	0.0670*** (12.5376)	0.0485*** (13.1185)	0.5575*** (25.1232)	0.6579*** (46.2360)
Lev	-0.0331 (-0.8334)	-0.0247 (-0.8191)	-0.1307 (-0.9796)	-0.4577*** (-4.2142)
Roe	0.0582 (1.0954)	0.1079*** (3.0964)	1.3912*** (7.3925)	0.3645*** (2.8278)
Grow	-0.0405** (-2.4658)	-0.0362*** (-2.8184)	0.0231 (0.4132)	0.1049** (2.3453)
Flow	-0.0056** (-2.3638)	-0.0092*** (-4.0382)	-0.0174** (-2.4208)	-0.0319*** (-4.4198)
Liqu	0.1582*** (4.5745)	0.1549*** (6.1452)	0.9490*** (7.4601)	0.7603*** (8.1059)
Top1	-0.0002 (-0.5796)	0.0003 (1.0449)	-0.0073*** (-5.8497)	-0.0039*** (-4.1603)
Dual	0.0020 (0.1895)	0.0028 (0.3676)	0.0608 (1.5685)	0.0254 (0.9127)
Deve	-0.0196 (-0.7393)	-0.0128 (-0.5666)	-0.3465*** (-3.7313)	0.0148 (0.1755)
Cons	-1.0704*** (-3.6692)	-0.6193** (-2.5627)	-7.9190*** (-7.4251)	-14.0899*** (-15.8028)
Industry	Yes	Yes	Yes	Yes
Year	Yes	Yes	Yes	Yes
Obs	4892	8432	4892	8432
Test for difference in coefficients between groups	P = 0.0224		P = 0.0133	
Adj_R^2^	0.1255	0.1260	0.2944	0.3490
**Panel B Level of development of the capital market sector**				
	**Market_Low**	**Market_High**	**Market_Low**	**Market_High**
Index	0.0254 (0.6531)	0.1050*** (2.7882)	0.2472** (1.9741)	0.6820*** (4.3443)
Size	0.0774*** (10.8821)	0.0434*** (9.6268)	0.4901*** (21.0503)	0.6464*** (31.8611)
Lev	-0.0571 (-1.5512)	-0.0477 (-1.1873)	-0.3900*** (-3.4027)	-0.4309*** (-2.6767)
Roe	0.0085 (0.1971)	0.1409*** (3.3203)	0.4353*** (3.2049)	0.9108*** (4.9748)
Grow	-0.0010 (-0.0544)	-0.0515*** (-3.5667)	0.0357 (0.6522)	-0.0109 (-0.1925)
Flow	-0.0080** (-2.3140)	-0.0171*** (-4.3343)	-0.0486*** (-4.8367)	-0.0700*** (-5.1554)
Liqu	0.2003*** (6.1390)	0.1071*** (3.6422)	0.8446*** (7.9197)	1.1735*** (9.4055)
Top1	0.0000 (0.0048)	0.0002 (0.7214)	-0.0061*** (-5.5761)	-0.0047*** (-4.1402)
Dual	-0.0213** (-2.1671)	0.0125 (1.2990)	-0.0584* (-1.8404)	0.0410 (1.0288)
Deve	-0.0038 (-0.1504)	-0.0892*** (-3.5405)	-0.2183*** (-2.7320)	-0.3313*** (-3.1868)
Cons	-1.3285*** (-4.4268)	0.1877 (0.7079)	-7.3014*** (-7.7754)	-11.0199*** (-9.9880)
Industry	Yes	Yes	Yes	Yes
Year	Yes	Yes	Yes	Yes
Obs	5837	5888	5837	5888
Test for difference in coefficients between groups	P = 0.1399		P = 0.0299	
Adj_R^2^	0.1008	0.1711	0.1867	0.3901

Note

***, **, and * denote, in turn, significance at the 1%, 5%, and 10% confidence levels; numbers in parentheses are t-values, and regression results are based on robust standard errors.

Combining the regression results of the grouping of digital finance on corporate breakthrough innovation under the conditions of different levels of development of the banking sector and the capital market sector, digital finance can play a greater role in corporate breakthrough innovation in provinces with a low level of development of the banking sector; however, in provinces with a high level of development of the capital market sector, digital finance plays a greater role in corporate breakthrough innovation. The lower the level of development of the banking sector and the higher the level of development of the capital market sector, the more room for digital finance to play a role. This suggests that digital finance has a gap-filling effect on the banking sector and an advantage-gaining effect on the capital market sector in promoting breakthrough innovation. This phenomenon may be because, on the one hand, digital finance can not only absorb more financial resources and transform them into effective supply with the help of digital technology [10], but also reshape the competitive landscape of the banking sector [58], promote the transformation and upgrading of the banking sector, and form an "incremental supplement" and "stock optimization" to traditional finance. Therefore, digital finance can play a greater role in regions with a low level of development of banking sector development. On the other hand, although digital finance relies on digital technology to realize the lack of traditional finance, technological innovation is also an important part of the source of financial risk [55]. The contagious characteristics of financial risk will cause this instability to be transmitted to the capital market. The perfect capital market has a stronger ability to withstand risk, which makes the higher level of development of the capital markets less affected by financial risk. Thus, digital finance can play a greater role in regions with higher capital market sector development levels.

#### 6.4.2 Regional disparities

China is a vast country with large differences in resource endowment and different levels of economic development between regions. The eastern region is at a higher level in terms of the quality of the institutional environment and the development of the capital market. Therefore, the uncertainty and risk of digital financial development in the process of universalization in the eastern region are lower, and the level of development is higher. However, on the other hand, due to the large space for development, the less developed regions in the central and western regions may be affected by digital financial development to a greater extent compared with the developed regions in the east. This shows that the academic community has not yet reached a consensus on the regional variability of the consequences of the impact of digital finance. In order to test whether there is regional variability in the impact of digital finance on the breakthrough innovation of enterprises, this paper divides the sample enterprises according to the region into the sample group of the eastern region (East), the sample group of the central region (Mid) and the sample group of the western region (West), and carries out a group regression on the benchmark model (2).

The regression results of the regional variability of digital finance in driving breakthrough innovation in firms are shown in Table 17. The regression coefficients of digital finance on the degree of dispersion of enterprise patent distribution and the number of invention patent applications are significantly positive at the 1% confidence level in the sample group of the central region, and positive but not significant in the sample groups of the eastern region and the western region. The test of difference in coefficients between groups shows that the regression coefficients of digital finance on breakthrough innovation of enterprises are significantly different in the sample group of the eastern region and the sample group of the central region, as well as in the sample group of the central region and the sample group of the western region. It shows that there is regional variability in the positive effect of digital finance on breakthrough innovation of enterprises, and the development of digital finance in the central provinces has a greater role in promoting breakthrough innovation of enterprises. The possible reason is that the eastern region has a high degree of marketization, developed capital market and high level of financial development, enterprises face lower financing constraints, the level of innovation is higher, and the space for digital finance to play is relatively small; in the central region, relative to the eastern region and the western region, the degree of marketization, capital market and financial development level are at the intermediate level, enterprises face more serious financing constraints, the level of innovation is relatively insufficient, and digital finance can play a larger space; the western region has a low level of economic development, the degree of marketization, the capital market and the level of financial development are in an underdeveloped state, enterprises face serious financing constraints, the level of innovation is low, the space for digital finance to play should be greater. However, on the other hand, the role of digital finance cannot be separated from the support of financial infrastructure, institutional environment, human resources, etc., and the western region is still immature in these institutions and mechanisms, resulting in digital finance not playing its due role.

**Table 17:** The regression results of regional disparities heterogeneity.

	Break			Patent		
	East	Mid	West	East	Mid	West
	(1)	(2)	(3)	(4)	(5)	(6)
Index	0.0036 (0.0963)	0.3701*** (4.8657)	0.1330 (1.2864)	0.0228 (0.1750)	1.1466*** (4.8578)	0.4457 (1.2982)
Size	0.0479*** (15.0423)	0.0682*** (9.5511)	0.0728*** (8.8208)	0.6558*** (51.9079)	0.5470*** (20.0339)	0.5913*** (17.2713)
Lev	-0.0408 (-1.5570)	-0.0443 (-0.8022)	-0.0252 (-0.3682)	-0.2129** (-2.2818)	-0.5250*** (-2.9228)	-0.7368*** (-3.1492)
Roe	0.1314*** (4.1369)	0.0994 (1.4398)	-0.0945 (-1.2585)	0.6478*** (5.5392)	0.4316** (1.9901)	0.4095 (1.5175)
Grow	-0.0226** (-2.0856)	-0.0602*** (-2.6137)	-0.0137 (-0.5262)	0.0734* (1.8827)	0.0671 (0.8931)	0.0914 (1.0416)
Flow	-0.0072*** (-4.1220)	-0.0147*** (-3.0405)	-0.0051 (-0.9350)	-0.0151*** (-2.7390)	-0.0784*** (-5.3846)	-0.0411** (-2.5663)
Liqu	0.1434*** (6.6290)	0.3262*** (6.6201)	0.1379** (2.5285)	0.7234*** (8.9050)	1.5722*** (8.3814)	0.6475*** (3.3070)
Top1	-0.0003 (-1.2319)	0.0015*** (3.2055)	0.0007 (1.2138)	-0.0043*** (-5.3748)	-0.0029 (-1.6064)	-0.0018 (-0.8365)
Dual	0.0044 (0.6968)	0.0161 (1.0100)	-0.0376 (-1.6057)	0.0222 (0.9537)	0.0834 (1.5488)	-0.0375 (-0.4884)
Deve	-0.0083 (-0.6538)	-0.0659** (-2.1886)	-0.0014 (-0.0398)	0.1276*** (2.8101)	-0.8818*** (-8.9285)	-0.2673** (-2.1321)
Cons	-0.6263*** (-5.5449)	-2.0200*** (-8.4583)	-1.8764*** (-4.9388)	-15.1946*** (-36.6578)	-6.6270*** (-7.6003)	-10.4964*** (-7.5726)
Industry	Yes	Yes	Yes	Yes	Yes	Yes
Year	Yes	Yes	Yes	Yes	Yes	Yes
Obs	11484	2507	1574	11484	2507	1574
Test for difference in coefficients between groups	P = 0.000			P = 0.000		
		P = 0.0621			P = 0.0893	
Adj_R^2^	0.1219	0.1715	0.1499	0.3556	0.3420	0.2992

Note

***, **, and * denote, in turn, significance at the 1%, 5%, and 10% confidence levels; numbers in parentheses are t-values, and regression results are based on robust standard errors.

#### 6.4.3 Enterprise scale

Financing constraints are essential to corporate breakthrough innovation, and enterprise scale can significantly impact financing constraints. Hottenrott and Peters [48] argued that financing constraints do not significantly affect the investment behavior of large-scale enterprises. In contrast, Cao et al. [59] pointed out that financing constraints constrain the innovation of small-scale enterprises. In order to test whether enterprise scale heterogeneity can have an impact on the facilitating effect of digital finance on corporate breakthrough innovation, this paper conducts a group regression of the benchmark model (2) by dividing the sample enterprises into a sample group of large-scale enterprises (Large) and a sample group of small-scale enterprises (Small) using the yearly median of the enterprise scale as a criterion.

The regression results of the enterprise scale heterogeneity of digital finance driving breakthrough innovation are shown in Table 18. In columns (1) and (3), the regression coefficients of digital finance on breakthrough innovation of large-scale enterprises are significantly positive at the 1% level, and in columns (2) and (4), the regression coefficients of digital finance on breakthrough innovation of small-scale enterprises are positive but not significant. The between-group coefficient difference test shows that digital finance promotes breakthrough innovation in large-scale enterprises more significantly than in small-scale enterprises. This may be due to large-scale enterprises being more willing to explore new technological fields because they have a specific innovation base and a more vital risk-taking ability.

**Table 18:** The regression results of enterprise scale heterogeneity.

	Break		Patent	
	Large	Small	Large	Small
	(1)	(2)	(3)	(4)
Index	0.1478*** (3.5535)	0.0301 (0.6984)	0.4931*** (3.1241)	0.0234 (0.1798)
Size	0.0416*** (10.0489)	0.0792*** (9.4489)	0.6704*** (36.2622)	0.5943*** (22.0850)
Lev	-0.0003 (-0.0080)	-0.0682** (-2.0751)	-0.4909*** (-3.9560)	-0.3324*** (-3.2378)
Roe	0.1459*** (4.0557)	0.0559 (1.3934)	0.9875*** (7.0816)	0.0827 (0.6418)
Grow	-0.0546*** (-4.4116)	0.0010 (0.0778)	-0.0034 (-0.0719)	0.1604*** (3.7709)
Flow	-0.0090*** (-2.6029)	-0.0071*** (-3.7414)	-0.0606*** (-4.7487)	-0.0176*** (-3.1798)
Liqu	0.1317*** (5.2581)	0.1834*** (6.4494)	1.0984*** (10.5028)	0.4426*** (4.8500)
Top1	0.0002 (0.9318)	-0.0001 (-0.3107)	-0.0045*** (-4.5136)	-0.0043*** (-4.4654)
Dual	0.0081 (0.9712)	0.0047 (0.6080)	0.0781** (2.3052)	-0.0066 (-0.2575)
Deve	-0.0717*** (-4.9123)	0.0277* (1.8227)	-0.0785 (-1.4141)	0.0336 (0.6791)
Cons	-0.4060*** (-2.9799)	-1.8296*** (-8.7364)	-15.5107*** (-29.0917)	-12.1181*** (-17.8941)
Industry	Yes	Yes	Yes	Yes
Year	Yes	Yes	Yes	Yes
Obs	7781	7784	7781	7784
Test for difference in coefficients between groups	P = 0.0488		P = 0.0213	
Adj_R^2^	0.1465	0.1005	0.3555	0.1400

Note

***, **, and * denote, in turn, significance at the 1%, 5%, and 10% confidence levels; numbers in parentheses are t-values, and regression results are based on robust standard errors.

## 7. Conclusions and implications

### 7.1 Conclusions

This paper takes A-share listed companies in Shanghai and Shenzhen from 2011 to 2022 as research samples to empirically analyze the impact effect and path of digital finance on breakthrough innovation of enterprises. The study finds that: (1) digital finance can promote breakthrough innovation of enterprises. The path test shows that digital finance promotes breakthrough innovation through alleviating financing constraints and expanding social networks. (2) There are differences in the impact of different dimensions of digital finance on the breakthrough innovation of enterprises, with the depth of the use of digital finance having the greatest impact on the breakthrough innovation of enterprises, the breadth of the coverage of digital finance having the second greatest impact on the breakthrough innovation of enterprises, and the degree of digital support services not showing any impact on the breakthrough innovation of enterprises. (3) The degree of enterprises’ external financing dependence and the degree of product market competition can strengthen the positive effect of digital finance on enterprises’ breakthrough innovation; the higher the degree of enterprises’ external financing dependence and the degree of product market competition, the greater the positive effect of digital finance on promoting enterprises’ breakthrough innovation. (4) The promotion effect of digital finance on breakthrough innovation of enterprises is heterogeneous in provinces with different levels of financial development; in provinces with a lower level of development of the banking sector and a higher level of development of the capital market sector, the promotion effect of digital finance on the breakthrough innovation of enterprises is greater; there are differences in the impact of digital financial development on the breakthrough innovation of enterprises in different regions; relative to the eastern and western regions, the impact of digital finance on the breakthrough innovation of enterprises in the central region is greater than that in the eastern and western regions. finance has a greater role in driving breakthrough innovation for firms in the central region.

### 7.2 Implications

In view of the above findings, the following policy insights are obtained: (1) In the context of the country’s active financial supply measurement reform, on the one hand, provinces and municipalities should continue to steadily promote the development of digital finance, actively play a role in optimizing the allocation of financial resources, improve the financing environment, alleviate the constraints on enterprise financing, enable digital finance to better serve the real economy, promote breakthrough innovations, and support the enterprises’ healthy and long-term development. On the other hand, enterprises should seize the golden period of the current development of digital finance, make good use of the social platform attributes of digital finance, expand their own social networks, strengthen cooperation with other enterprises, acquire heterogeneous knowledge and cutting-edge technologies, actively carry out breakthrough innovation activities, improve the quality of innovation and enhance innovation capability. (2) In encouraging the integration of IT and financial markets and promoting the development of digital finance, provinces and cities should, on the one hand, continue to expand the coverage of financial services, give full play to the inclusive nature of digital finance, try to eliminate discrimination in innovation financing, promote the equalization of financial resource allocation, create a fair, inclusive and open innovation environment, and unleash the vitality of enterprise innovation. On the other hand, on the basis of the expanding coverage of digital finance, the depth of digital financial use should be enhanced by popularizing financial knowledge, and the degree of digital support services should be deepened by supporting the combination of “Internet Plus” and the financial industry, so as to realize the coordinated development of digital finance in multiple dimensions. (3) To further improve and supplement the financial system, on the one hand, in areas with a low level of development of the banking sector, traditional financial institutions should actively embrace the development trend of digital finance, utilize digital technology to effectively screen enterprises, and give sufficient financial support to enterprises with strong innovation ability, so as to play the role of digital finance in supplementing the shortcomings of the banking sector; on the other hand, to comprehensively develop the capital market, improve the level of development of the capital market, and effectively play the role of the capital market in the development of the digital finance sector. On the other hand, comprehensively develop the capital market, enhance the development of the capital market, effectively utilize the advantageous gain effect of the capital market in the process of digital finance to promote enterprises to make breakthrough innovations, and help the implementation of the innovation-driven development strategy. (4) Reform the current financial regulatory system to balance the relationship between digital financial development, financial risks, and innovation in the real economy. On the one hand, regulators should implement sustained and focused policies to stabilize market expectations. On the other hand, the use of digital technology, artificial intelligence, and other scientific and technological means to build a regulatory technology system, to enhance the relevance, immediacy, and penetration of regulatory technology, to effectively guide digital finance to inject kinetic energy into the development of innovation, and to prevent financial risks and other chaotic phenomena that may be induced in the process of its development.

### 7.3 Limitations and future outlooks

Although this paper has some marginal contributions, there are still some limitations: (1) This paper explores the role mechanism of digital finance affecting breakthrough innovation of enterprises from the perspective of financing constraints and social networks, but there may be other role paths, and in the future, a more precise and specific theoretical analytical analysis framework can be established to more comprehensively understand the impact of digital finance on corporate breakthrough innovation. (2) This paper mainly examines the impact of digital finance on corporate breakthrough innovation, but the impact on firm value and core competitiveness has not yet been explored in depth, and in the future, the research on digital finance and corporate breakthrough innovation can be extended to comprehensively assess the positive effects of digital finance on micro-enterprises.

## Supporting information

S1 Data (XLSX)
